# Electrochemical, surface morphological and computational evaluation on carbohydrazide Schiff bases as corrosion inhibitor for mild steel in acidic medium

**DOI:** 10.1038/s41598-023-41975-9

**Published:** 2023-09-13

**Authors:** Sujata Kumari Gupta, R. K. Mitra, Mahendra Yadav, Omar Dagdag, Avni Berisha, Bhekie B. Mamba, Thabo T. I. Nkambule, Eno E. Ebenso, Shailendra Kumar Singh

**Affiliations:** 1 Department of Chemistry and Chemical Biology, Indian Institute of Technology (ISM), Dhanbad, Jharkhand 826004 India; 2 Centre for Materials Science, College of Science, Engineering and Technology, University of South Africa, Johannesburg, 1710 South Africa; 3 Institute of Nanotechnology and Water Sustainability, College of Science, Engineering and Technology, University of South Africa, Johannesburg, 1710 South Africa; 4 Department of Chemistry, Faculty of Natural and Mathematics Science, University of Prishtina, 10000 Prishtina, Kosovo; 5 Department of Chemistry, Hansraj College, University of Delhi, Delhi, 110007 India

**Keywords:** Chemistry, Materials science

## Abstract

Anticorrosion and adsorption behaviour of synthesized carbohydrazide Schiff bases, namely (Z)-*N*′-(4-hydroxy-3-methoxybenzylidene)-6-methyl-2-oxo-4-phenyl-1,2,3,4-tetrahydropyrimidine-5-carbohydrazide(MBTC) and (Z)-*N*′-(3,4-dichlorobenzylidene)-6-methyl-2-oxo-4-phenyl-1,2,3,4-tetrahydropyrimidine-5-carbohydrazide (CBTC) was examined for mild steel (MS) in 15% HCl medium. The corrosion inhibition study was performed by using gravimetric, thermodynamic, electrochemical and theoretical studies including density functional theory (DFT), molecular dynamic simulation (MDS) and Monte Carlo simulations (MCS). The outcomes in terms of corrosion inhibition efficiency using electrochemical impedance spectroscopy (EIS) method at 303 K and 150 ppm concentration were 96.75% for MBTC and 95.14% for CBTC. Both inhibitors adsorbed on the MS surface through physical as well as chemical adsorption and followed the Langmuir isotherm. The mixed-type nature of both inhibitors was identified by polarization results. Surface analysis was done using FESEM, EDX, AFM and XPS studies and results showed that a protective layer of inhibitor molecules was developed over the surface of MS. The results of DFT, MCS and MDS are in accordance with experimental results obtained by weight loss and electrochemical methods.

## Introduction

The research on corrosion inhibition is considered as a challenging problem because corrosion is spontaneous and continuous degradation of metallic structures using electrochemical and chemical reactions with the surrounding, which cause severe safety and economic loss in various sectors^1^. The mild steel (MS) is considered as core structural element in many industries due to its effective potency in flexibility and cheaper cost. Many industries use solution of HCl and H_2_SO_4_ for acid pickling of steel and iron, production of ore, oil well acidization and removal of rust as well as scale. During these operations, due to the corrosive properties of acid solution, severe corrosion of metal happens which creates environmental as well as economic issues^2,3^. To minimize this loss, various corrosion prevention techniques like coating, selection of alloy, galvanization, cathodic and anodic protections, and selection of inhibitors are used. From these methods, addition of corrosion inhibitors in the solution phase is the most useful techniques for prevention of corrosion^4^. The adsorption of corrosion inhibitors on MS surface depends on the structure of corrosion inhibitor and played a significant role in the formation of corrosion protecting film on the surface of MS. The addition of small amount of the corrosion inhibitor provides rise in efficiency by instantly decreasing the corrosion rate^5^. In application of corrosion inhibitors, industries are facing challenge because most of the corrosion inhibitor molecules are toxic and harmful for both human and environment. The compounds containing functional groups such as NH_2_, OH, OCH_3_, C=N, C=O, SH, and hetero atoms like O, N, P, S as well as unsaturated aromatic system work as efficient inhibitors^6–12^. Schiff bases showed excellent inhibition efficiency for different metals and alloys such as copper, brass, aluminum, MS, and stainless steel in different corrosive mediums. Presently Schiff bases are used as inhibitor because of their simple synthetic procedure, low-cost, high-yield and less toxic properties. Apart from these, Schiff base compounds are also used in catalysis, photochromic field, and medicine^13^. Because of easy synthesis, high efficiency and low toxicity, Schiff base compounds are good choice in selection of inhibitors and various researchers have used Schiff base compounds as corrosion inhibitor^14,15^. The Schiff base compound reported as corrosion inhibitor in literature for MS in HCl medium^16–30^ are represented in Table 1.

**Table 1:** Summary of already reported Schiff bases as corrosion inhibitors for MS in HCl solution.

S. no	Inhibitor	Medium	Material	Inhibition efficiency (η %)	Conc. (ppm)	Ref
1	Potassium-(2-(((1H-indol-3-yl)methylene)amino)acetyl) tyrosinate [GTI]	1 M HCl	Mild Steel	98.70	403	16
2	*N*,*N*′-(1,3-phenylene)bis(1-(pyridin-2-yl)methanimine) [PBPM]	1.0 mol^−1^ HCl	Mild Steel	78.35	800	17
3	4-((2,3-dichlorobenzylidene) amino) -3-methyl-1H-1,2,4-triazole-5(4H)-thione, [CBAT]	1 M HCl	Mild Steel	95.00	287	18
4	*N*,*N*′-(1,4-phenylene)bis(1-(pyridin-3-yl)methan-imine) [PBPM3]	1 M HCl	Mild Steel	83.92	800	19
5	(1E,2E)-*N*-(3-(1H-imidazol-1-yl)propyl)-3-phenylprop-2-en-1-imine [ICSB]	1 M HCl	Mild Steel	98.80	239	20
6	2-((E)-((E)-2-hydrazone-1,2-diphenylethylidene)amino phenol) [HDAP]	1 M HCl	Mild Steel	92.76	3153	21
7	4,4′-Oxybis *N*-[(E)quinoleine-2-ylmethylidene]aniline[OQ]	1 M HCl	Mild Steel	86.46	2392	22
8	Vanillin cyanoguanidine imine [VCNG]	1 M HCl	Mild Steel	93.10	499	23
9	2-(2-oxoindolin-3-ylidene) hydrazinecarbothioamide [OHB]	1 M HCl	Mild Steel	96.00	220	24
10	(E)-4-(1-(pyridin-2 ylimino) ethyl)benzene-1,3-diol [PSB2]	1 M HCl	Mild Steel	90.00	228	25
11	3,3'-dimethoxy-5,5'-bis((E)-((pyridin2ylmethyl)imino)methyl)-[1,1'-biphenyl]-2,2'-diol [vanillin based]	1 M HCl	Mild Steel	95.30	482	26
12	4,4’-bis(2-furylideneimino)biphenyl]-4,4'-diamine [HL2)]	1 M HCl	Mild Steel	89.41	340	27
13	*N*-(2-((Z)-2-(3-methoxy-4-hydroxybenzylideneamino) ethylamino)ethyl)-3,4,5-trihydroxybenzamide [VA]	0.5 M HCl	Mild Steel	88.52	250	28
14	*N*-[(Z)-1-phenylemethyleidene]-*N*-{2-[(2-{[(Z)-1-phenylmethylidene]amino}phenyl)disulfanyl]phenyl}amine	2.0 M HCl	Mild Steel	96.00	424	29
15	(1,2-1H-Benzoimidazol-2-yl)-(1,2-diphenyl-ethylidene)-amine) [BDEA]	1.0 M HCl	Mild Steel	96.85	400	30

Inspired with the use of Schiff base compounds as efficient and low toxic corrosion inhibitors and with expectation of higher inhibition efficiency as compared to reported inhibitors, in the present investigation carbohydrazide Schiff base compounds, (Z)-*N*′-(3,4-dichlorobenzylidene)-6-methyl-2-oxo-4-phenyl-1,2,3,4-tetrahydropyrimidine-5-carbohydrazide(CBTC)and (Z)-*N*′-(4-hydroxy-3-methoxybenzylidene)-6-methyl-2-oxo-4-phenyl-1,2,3,4-tetrahydropyrimidine-5-carbohydrazideon (MBTC) were prepared for corrosion inhibition study of MS in 15% HCl. The selection of Schiff bases was done according to their planar structure and good molecular framework which promotes better inhibition efficiency. Two Schiff bases were taken with the same moiety, but different substitution groups to study the effect of substituent’s on corrosion inhibition behavior of the inhibitors.

## Experimental procedure

### Synthesis of carbohydrazide Schiff base inhibitors

**Figure 1:**
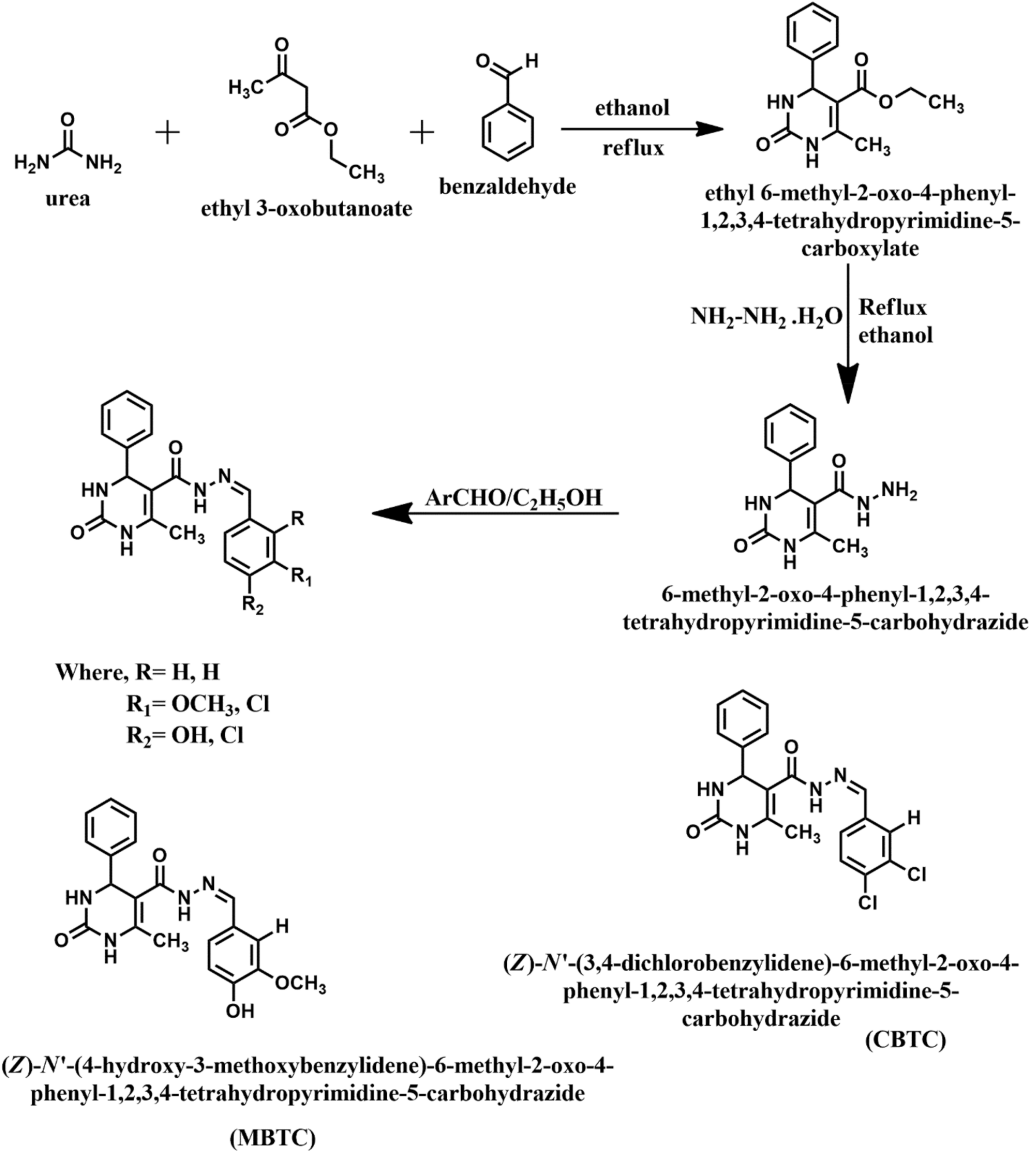
Reaction scheme of MBTC and CBTC synthesis.

Both the inhibitors were prepared in the laboratory as per reported procedure^31^ as shown in Fig. 1. The mixture of urea (0.15 mol), ethyl acetoacetate (0.1 mol), benzaldehyde, ethanol (25–30 mL) and conc. HCl in 3-drops was refluxed for 1:30 h. After completion of the reaction, reaction mixture was put into ice cold water with continuous stirring, then it was kept for 18 h, mixture was filtered and dried. The compound formed was called *biginelli*, it was recrystallized through ethanol and tested by thin layer chromatography. After recrystallization, mixture of *biginelli* (0.01 mol), hydrazine hydrate, ethanol (approx. 20–22 mL) and 4-drops of conc. H_2_SO_4_ was refluxed for 3 h. After completion of reaction, the mixture was recrystallized through ethanol and the product formed was called carbohyrazido compound. The mixture of carbohyrazido (0.01 mol), substituted aromatic aldehyde, ethanol (20 mL) and 5 mL of glacial acetic acid was refluxed for 2 h. Upon the accomplishment of reaction, it was placed into cold water, the product was filtered and dried. TLC was used to test the purity of the compound, the precipitate was recrystallized using ethanol and the obtained compounds are MBTC and CBTC. Both compounds were characterized and obtained data are as follows:

#### MBTC

Mol. Formulae: C_20_H_20_N_4_O_4,_ Mol. Weight: 380.40, Yield: 78%, Elemental analysis: calculated %weight- C: 63.15, N: 14.73, H: 5.30, O: 16.82.

FTIR, (cm^−1^) 3551.75(NH), 3484.26(OH), 3244.16(NH in amide), 3124.6 (AR–CH), 2931.75 (Aliphatic CH), 1700.91(C=O in amide), 1648.36 (C=O), 1091.99 (C–O–C).

#### CBTC

Mol. Formulae: C_19_H_16_Cl_2_N_4_O_2_, Mol. Weight: 403.26, Yield: 80%, Elemental analysis: calculated %weight- C: 56.59, N: 13.89, O: 7.93, Cl: 17.58, H: 4.00.

FTIR (cm^−1^) 3421.58(NH), 3244.16 (NH in amide), 3114.47 (Ar–CH), 2937.06 (Aliphatic CH), 1700.91 (C=O in amide), 781.99 (C–Cl).

### Material

All investigations were conducted using MS with elemental percentage composition, as; Sn: 0.03, Ni: 0.02, Mn: 0.13, Cu: 0.01, P: 0.02, C: 0.13 along with Fe (iron: rest elemental percentage). MS was cut into the dimension of 3.5 cm × 3.0 cm × 0.1 cm for gravimetric method and for electrochemical analysis 1.0 cm × 1.0 cm × 0.1 cm individually. Several grades (i.e., 80 to 2000) of emery paper were used to clean the MS surface before each experiment successively so that homogeneous surface was obtained. These samples were then thoroughly cleaned in an ultrasonic cleaner for 15 min, cleaned with acetone, and dried. The solution of inhibitor concentrations ranging from 0 to 150 ppm was made in 15% HCl by dilution of analytical grade 37% HCl with distilled water.

### Gravimetric Analysis

Gravimetric analysis was used to investigate how temperature and concentration affected the examined Schiff base ability to prevent corrosion. MS specimens were submerged in 500 mL 15% HCl solution for 6 h at different temperatures ranging from 303 to 333 K, without and with several concentration of synthesized carbohydrazide Schiff base inhibitors. The specimens were weighed before and after submersion and weight loss was calculated. Each experimental value was calculated from the average data of triplicate value of weight loss^32^.The corrosion rate (CR), efficiency ($\eta \%)$ and surface coverage ($\theta )$ were calculated as follows:

1 $${CR (mm year)}^{-1}=\frac{W*8.76*{10}^{4}}{T*D*A}$$

where average weight loss is represented by W, sample area by A, immersion time by T (in hours), and sample density by D (in gm cm^−3^). The efficiency (η %) along with surface coverage (θ) of the investigated inhibitor are calculated by utilizing the Eqs. (2) and (3), as below:

2 $$\eta \%=\frac{{CR}_{0}-{CR}_{I}}{{CR}_{0}}*100$$

3 $$\theta =\frac{\eta \%}{100}$$

where ${CR}_{0}$ and ${CR}_{I}$, stands for corrosion rates without and with synthesized carbohydrazide Schiff base inhibitors.

### Electrochemical analysis

Electrochemical analysis was executed in conventional 3-electrode system cell by the electrochemical corrosion analyzer (CS-350) monitoring through CS Studio. Saturated calomel electrode act as the reference electrode, platinum electrode serves as the counter electrode and MS with surface area of 1cm^2^ serve as working electrode. Before starting the experiment, the cells’ working electrode is maintained in contact with 200 mL of acidic solution for 1800s for attaining steady state condition. Later potentials were examined in the range of ± 2.5V with a scan rate of at 0.5 mV s^−1^ from OCP which provides the polarization curves. Subsequently, at an open circuit voltage and frequency range of 100 kHz to 1Hz, electrochemical impedance spectroscopy (EIS) was evaluated^33^.

### Surface analysis

For surface analysis the MS specimen was prepared by pervious procedure as described from weight loss analysis. Before and after adding 150 ppm of inhibitor at 303 K, the MS surface was submerged for 6 h in 15% HCl medium. The specimen was removed after 6 h and washed with distilled water and then dried and further sampled for FESEM, EDX, AFM and XPS analysis^34^.

### Computational studies

#### Quantum chemical calculations

All quantum chemistry calculations have been implemented with full geometric optimizations utilizing the Dmol3 module integrated into the Biovia Materials studio software. The geometric structures of the studied molecules were attained through global optimizations characterized by a calculation of the vibration frequencies using the DFT method by utilizing Dmol3, including the B3LYP/DND/COSMO (water) model^34–40^.

#### MC and MD simulations

Materials Studio 8.0, programmed created by Accelrys Inc, was used to run MC and MD^41–44^. A three-dimensional geometry simulation box whose dimensions are (24.823752 × 24.823752 × 18.241658) was used for simulations of the interaction between the MBTC along with CBTC molecules and the surface of Fe (110). The MDS were worked at a temperature of 298 K and the motion equations were integrated under the NVT canonical ensemble utilizing the COMPASS force field, with a time step of 1 fs and a simulation time of 800 ps. This model included Fe layer, 15 hydronium, 15 chloride, 700 H_2_O molecules and 1 inhibitor molecule and a 35 vacuum layer were involved in the simulation box.

## Results and discussion

### Gravimetric analysis

**Figure 2:**
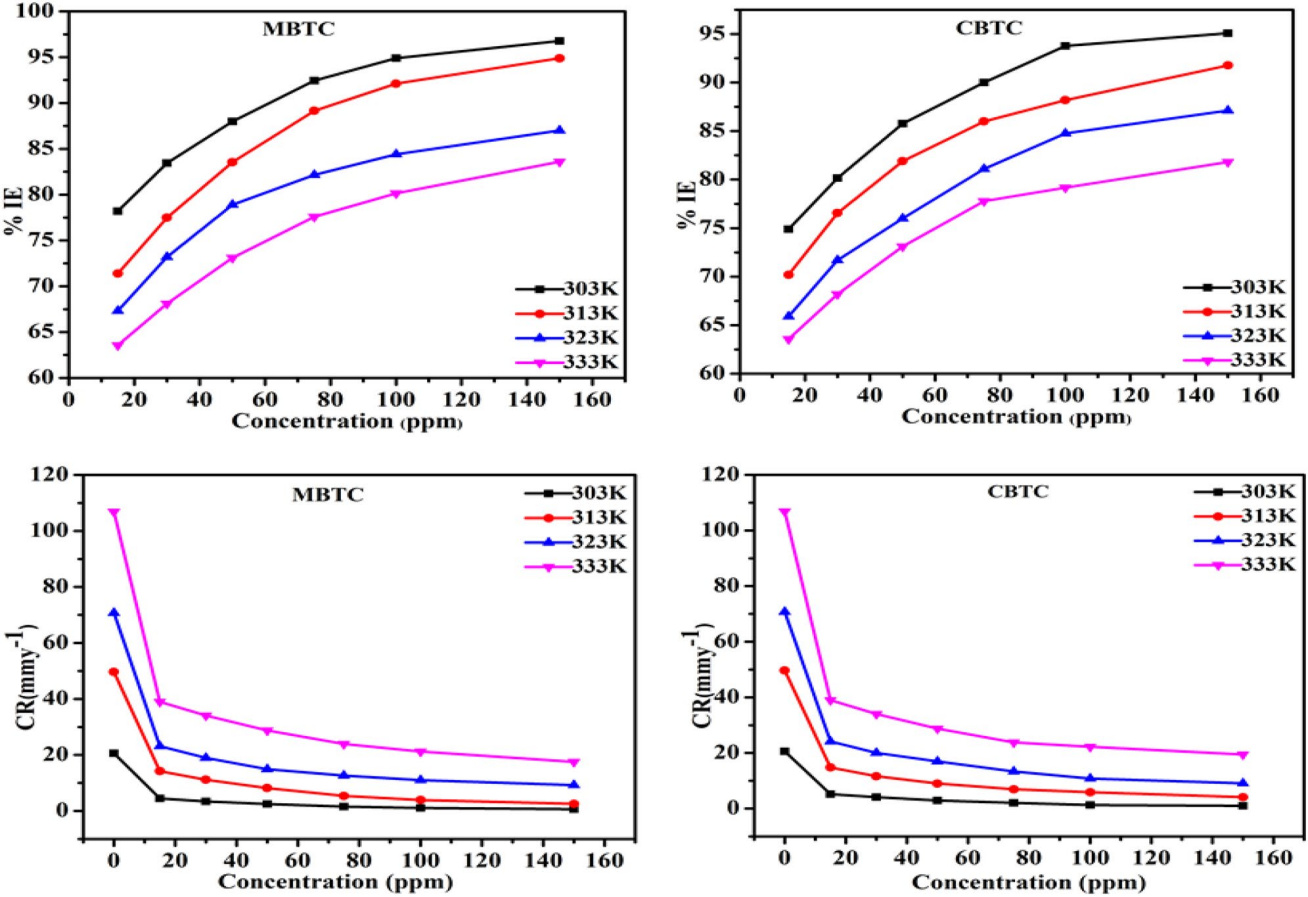
Plot of η % and (CR) at different temperatures for carbohydrazide Schiff base (MBTC and CBTC) inhibitor.

The gravimetric analysis is a first step in the investigation of corrosion inhibition. The effect of concentration on the inhibition efficiency (η %) and corrosion rate (CR) for the MS in 15% HCl at various temperatures (303 K–333 K) was done and obtained corrosion parameters are specified in Table 2. According to Table 2 and Fig. 2, it is found that, as the surface coverage of MS grew, the CR gradually dropped and corrosion inhibition (η %) gradually increased with rising inhibitor concentration. This indicated that sample surface is protected through the molecule of inhibitors during the increase in concentration as they are absorbed on the MS surface. Thus, the highest inhibitory efficiency obtained was at 150 ppm of MBTC as 96.78%, and it was 95.09% for CBTC. The higher efficiency of MBTC is because of the presence of electron donor group –OCH_3_, imine, carbonyl groups and heterocyclic rings (Fig. 1) which improved the efficiency level in the transfers of unpaired electrons from inhibitor molecules to the vacant d-orbital of Fe, which develops a coordinate-bond and thus slows the rate of corrosion (CR)^45^. The decrease in the inhibition potency on increase in temperature was observed due to removal of adsorbed inhibitory layer from MS surface. As per literature review, corrosion inhibition studies of MBTC and CBTC is not reported and both inhibitors offered better efficiency than most of the similar type of inhibitors reported in literature^16–30^ as shown in Table 1. Therefore the work reported in the present investigation is novel and has potential industrial application.

**Table 2:** Gravimetric parameters of carbohydrazide Schiff base inhibitor.

Conc	303 K			313 K			323 K			333 K		
	(CR)	(θ)	η %	(CR)	(θ)	η %	(CR)	(θ)	η %	(CR)	(θ)	η %
	mm y^−1^			mmy^−1^			mmy^−1^			mmy^−1^		
0	20.60	–	–	49.64	–	–	70.24	–	–	106.87	–	–
MBTC												
15	4.492 ± 0.016	0.781	78.19 ± 0.080	14.20 ± 0.166	0.713	71.38 ± 0.336	23.11 ± 0.154	0.673	67.32 ± 0.220	38.95 ± 0.093	0.635	63.55 ± 0.087
30	3.409 ± 0.056	0.834	83.45 ± 0.027	11.17 ± 0.057	0.774	77.48 ± 0.115	18.95 ± 0.017	0.732	73.20 ± 0.172	34.09 ± 0.364	0.681	68.10 ± 0.341
50	2.474 ± 0.118	0.879	87.99 ± 0.057	8.165 ± 0.105	0.835	83.55 ± 0.212	14.93 ± 0.017	0.788	78,89 ± 0.406	28.73 ± 0.234	0.731	73.11 ± 0.219
75	1.555 ± 0.038	0.924	92.45 ± 0.018	5.385 ± 0.084	0.891	89.15 ± 0.170	12.62 ± 0.021	0.821	82.15 ± 0.266	23.94 ± 0.431	0.775	77.59 ± 0.404
100	1.052 ± 0.083	0.948	94.89 ± 0.040	3.916 ± 0.067	0.921	92.11 ± 0.135	11.03 ± 0.024	0.844	84.40 ± 0.28	21.21 ± 0.564	0.801	80.15 ± 0.527
150	0.6633 ± 0.030	0.967	96.78 ± 0.014	2.536 ± 0.035	0.948	94.89 ± 0.070	9.196 ± 0.198	0.87	87.00 ± 0.282	17.53 ± 0.119	0.835	83.59 ± 0.111
200	0.5253 ± 0.048	0.974	97.45	2.233 ± 0.020	0.955	95.55 ± 0.413	8.849 ± 0.113	0.879	87.99 ± 0.13	16.15 ± 0.845	0.848	84.88 ± 0.532
CBTC												
15	5.172 ± 0.011	0.748	74.89 ± 0.055	14.79 ± 0.341	0.701	70.19 ± 0.688	24.12 ± 0.302	0.659	65.90 ± 0.430	38.95 ± 0.351	0.635	63.55 ± 0.328
30	4.089 ± 0.047	0.801	80.15 ± 0.229	11.64 ± 0.405	0.765	76.55 ± 0.816	20.01 ± 0.653	0.717	71.70 ± 0.929	33.99 ± 0.312	0.681	68.19 ± 0.292
50	2.931 ± 0.098	0.857	85.77 ± 0.479	8.984 ± 0.221	0.819	81.90 ± 0.446	16.97 ± 0.311	0.760	76.00 ± 0.443	28.74 ± 0.107	0.731	73.10 ± 0.100
75	2.06 ± 0.077	0.90	90.00 ± 0.377	6.949 ± 0.186	0.860	86.00 ± 0.376	13.37 ± 0.100	0.810	81.09 ± 0.142	23.75 ± 0.412	0.777	77.77 ± 0.385
100	1.283 ± 0.052	0.937	93.77 ± 0.256	5.862 ± 0.109	0.881	88.19 ± 0.221	10.77 ± 0.161	0.847	84.77 ± 0.229	22.25 ± 0.293	0.791	79.18 ± 0.274
150	1.011 ± 0.036	0.950	95.09 ± 0.176	4.085 ± 0.505	0.917	91.77 ± 1.017	9.111 ± 0.160	0.871	87.12 ± 0.228	19.42 ± 0.119	0.818	81.82 ± 0.111
200	0.8466 ± 0.030	0.958	95.89 ± 0.146	3.430 ± 0.146	0.930	93.09 ± 0.295	7.852 ± 0.561	0.889	88.90 ± 0.547	18.29 ± 0.835	0.828	82.88 ± 0.527

### Kinetic and thermodynamic activation parameters

**Figure 3:**
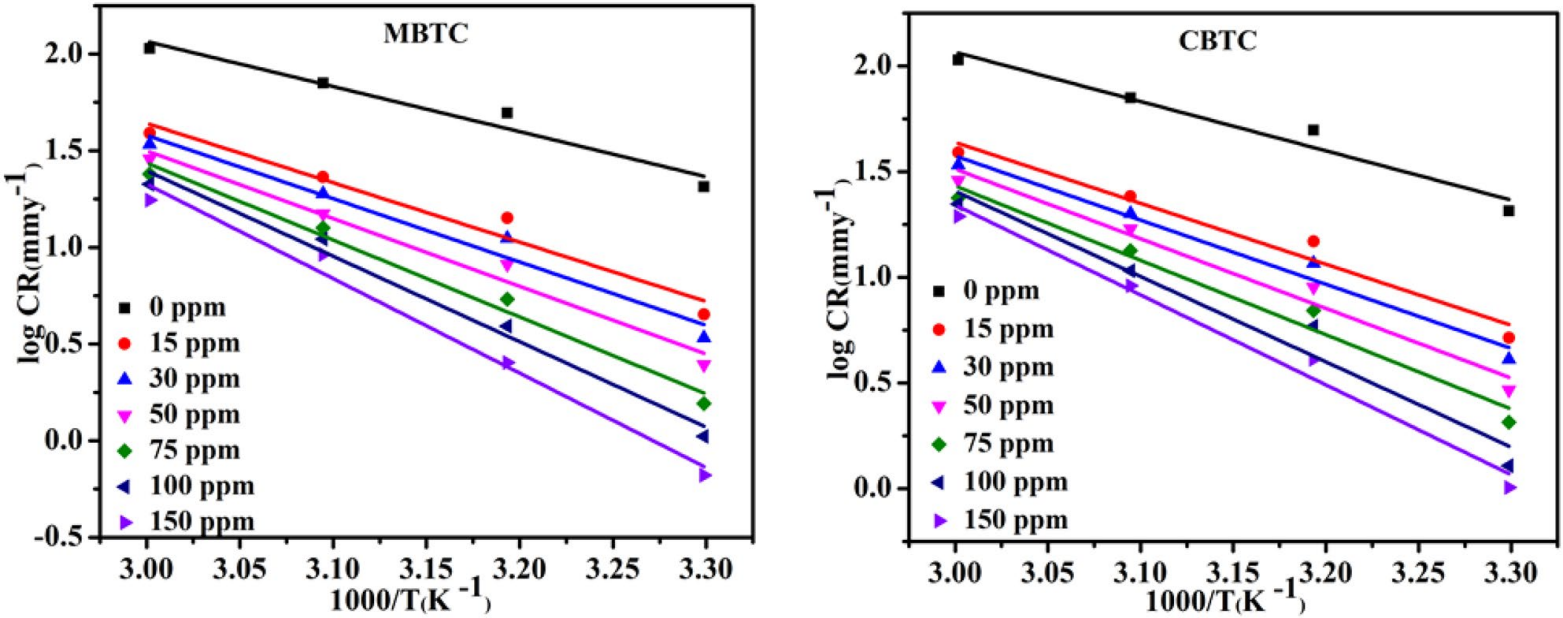
MBTC and CBTC Arrhenius plot for carbohydrazide Schiff base.

**Figure 4:**
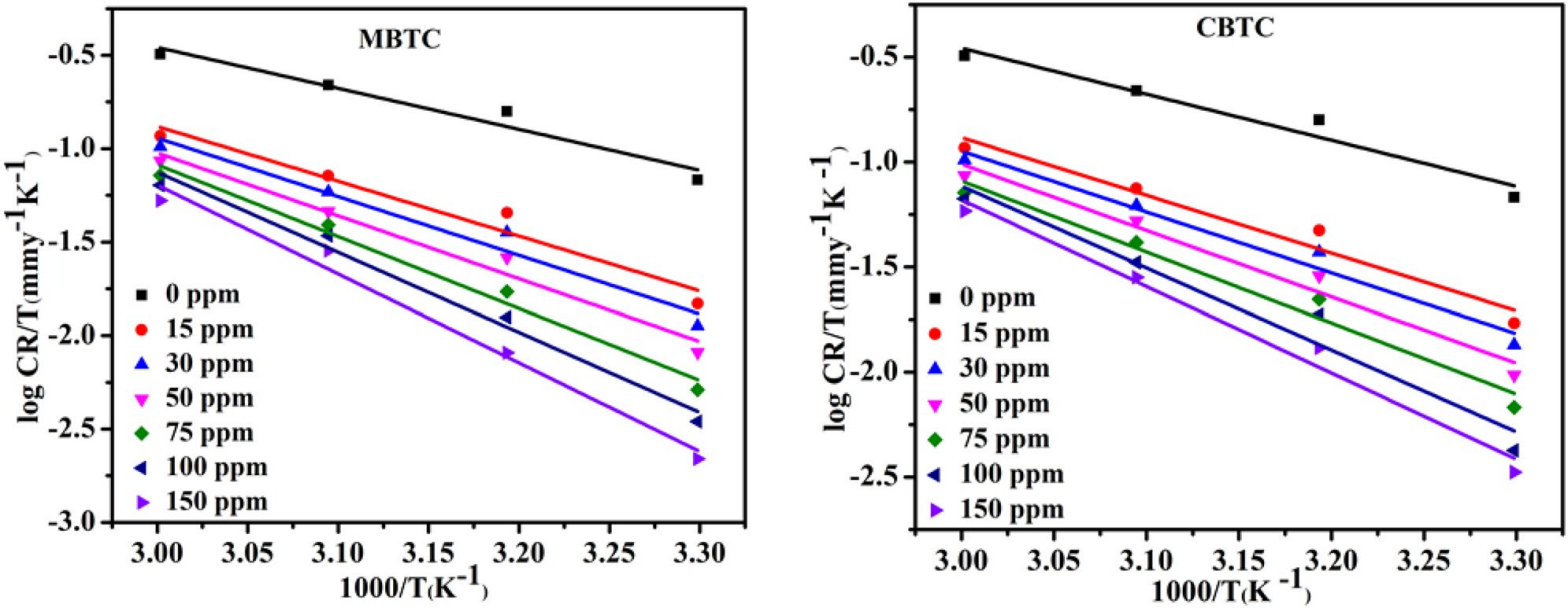
MBTC and CBTC Transition plot for carbohydrazide Schiff base.

The kinetic and thermodynamic parameters were obtained from Arrhenius and Transition Eqs. 4 and 5 and plots Figs. 3 and 4 as follows:

4 $$\mathrm{log}\left(CR\right)= \frac{{-E}_{a}}{2.303 RT}+\mathrm{log}A$$

5 $$CR=\left(\frac{RT}{NH}\right)expo\frac{{\Delta S}^{*}}{R}expo \frac{{-\Delta H}^{*}}{RT}$$

where activation enthalpy and entropy are represented by ∆H^∗^ and ∆S^∗^ while A is the pre-exponential component called Arrhenius constant respectively. T refers to the absolute temp., and H, N, R, refers to Planck constant, Avogadro number and gas constant respectively. By fitting the Arrhenius and transition plots shown in Figs. 3 and 4 respectively, the values for the activation parameters (${E}_{a}$) and ∆H^∗^ and ∆S^∗^ were determined and provided in Table 3.

**Table 3:** Thermodynamic and kinetic parameters of carbohydrazide Schiff base inhibitor.

Inhibitor	Concentration (ppm)	Ea (kJ mol^−1^)	∆H* (kJ mol^−1^)	∆S* (Jmol^−1^ K^−1^)
Blank	0	44.67	42.02	− 80.23
MBTC	15	58.80	56.15	− 45.94
	30	62.72	60.08	− 35.35
	50	67.09	64.44	− 23.79
	75	76.30	73.65	2.626
	100	84.66	82.02	26.94
	150	93.61	90.96	52.48
CBTC	15	55.23	52.59	− 56.68
	30	58.13	55.48	− 49.22
	50	63.12	60.48	− 35.43
	75	67.37	64.73	− 24.19
	100	77.33	74.69	5.15
	150	81.47	78.82	16.35

The ${E}_{a}$ value of inhibitors MBTC and CBTC for all concentrations are greater than uninhibited value. These demonstrate that a shielding layer was created on the MS surface and makes more resistant to corrosive process. From Table 3, the negative values of ∆S* reflect that the rate-determining step is the association rather than dissociation of activated complex, and the positive data of ∆S* denotes an increment in disorder because desorption of more number of water molecules from the surface of MS by adsorption of one molecule of inhibitor while the positive values of ∆H indicate the difficulty in MS dissolution^46^.

### Adsorption isotherm

Adsorption isotherm studies provide information on the interactions between the inhibitor and the MS surface. The following equation is representation of the Langmuir isotherm,

6 $$\frac{{C}_{inh}}{\theta }=\frac{1}{{K}_{ads}}+{C}_{inh}$$

**Figure 5:**
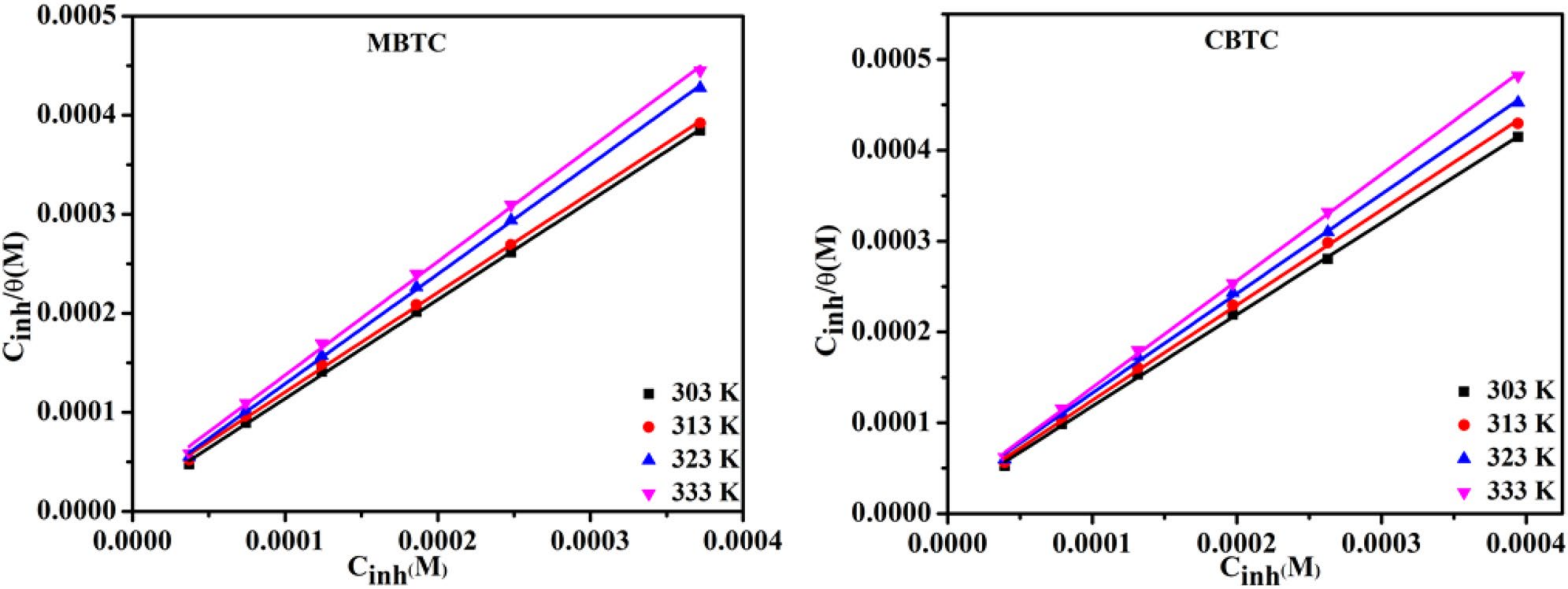
Langmuir plot for MBTC and CBTC inhibitors.

Different adsorption models were tried with the experimental data, Langmuir isotherm gave a linear plot between $\frac{{C}_{inh}}{\theta } vs$ Concentrations which are shown in Fig. 5, through which ${K}_{ads}$ value was calculated. Using ${K}_{ads}$ value, ${\Delta G}_{ads}^{0}$ value was derived from the following Eq. 7.

7 $${\Delta G}_{ads}^{0}=-RTln\left({55.5K}_{ads}\right)$$

R, T and 55.5 denote the gas constant, absolute temp., and the conc. (mol l^−1^) for water.

Since the R^2^ as well as slope values for the Langmuir isotherm are very close to one, it has been verified to be a superior fit for the examined value displayed in Table 4**.**

**Table 4:** Adsorption data of carbohydrazide Schiff base inhibitors.

Inhibitors	Temperature K	Slope	${K}_{ads}$ M^−1^	${\Delta G}_{ads}^{0}$ kJmol^−1^	R^2^
MBTC	303 K	0.99893	7.14E+04	− 38.27	0.99956
	313 K	1.00586	5.51E+04	− 38.61	0.99955
	323 K	1.10702	5.05E+04	− 40.10	0.99919
	333 K	1.14607	4.35E+04	− 40.69	0.99856
CBTC	303 K	1.00979	5.80E+04	− 37.74	0.99934
	313 K	1.04858	2.80E+04	− 38.63	0.99920
	323 K	1.09589	2.61E+04	− 39.45	0.99935
	333 K	1.17501	2.40E+04	− 40.90	0.99879

Experimental data from Table 4, has larger value of ${K}_{ads}$ indicating superior inhibition efficiency and good interaction between carbohyrazide Schiff base and MS surface whereas lower value of ${K}_{ads}$ indicates weaker interaction so that adsorbed molecules can be easily removed from MS surface.

The negative ${\Delta G}_{ads}^{0}$ values suggests that the adsorption of carbohydrazide Schiff base MBTC and CBTC on the surface of MS was proceeding spontaneously and the value of ${\Delta G}_{ads}^{0}$ obtained for both inhibitors verified physisorption as well as chemisorption adsorption at MS surface. According to the review of literature, ${\Delta G}_{ads}^{0}$ value less negative than − 20 kJ mol^−1^ results physisorption whereas more negative than − 40 kJ mol^−1^ results chemisorptions. Table 4 shows that the values of ${\Delta G}_{ads}^{0}$ for synthesized carbohydrazide Schiff base in 15% HCl at various temperatures attain the range showing the spontaneous chemisorption and physisorption, which is a mixed nature of adsorption^47^.

### Electrochemical analysis

#### Open circuit potential

**Figure 6:**
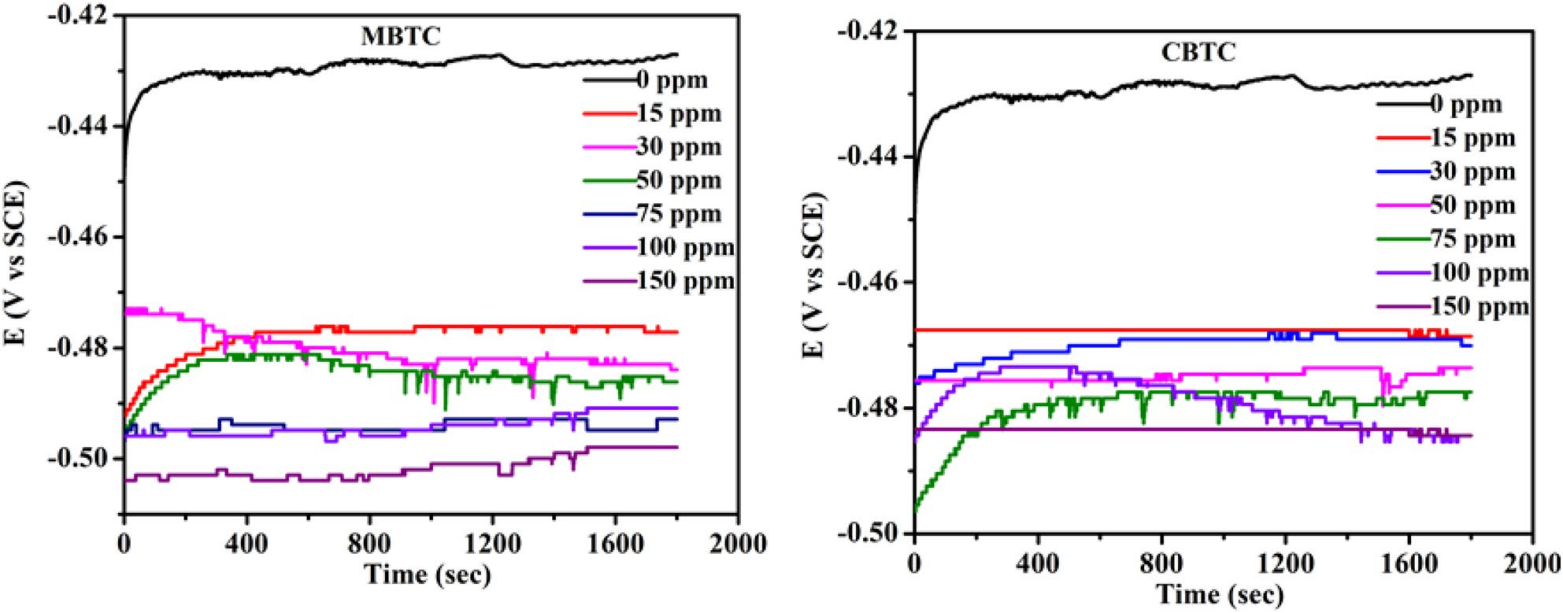
OCP plot for MBTC and CBTC carbohydrazide Schiff base inhibitors.

Figure 6 reveals the variation of OCP w.r.t. time in the absence and presence of different dosages of carbohydrazide Schiff base inhibitors at 303 K. Figure 6, displays that the addition of MBTC and CBTC inhibitors shifted the potential to the negative side when compared with pure 15% hydrochloric acid corrosive medium, indicating that both inhibitors are predominantly cathodic. Whereas the obtained data of OCP for the working electrode with various dosages of both inhibitors alter OCP to an extent of less than $\pm$ 85 mV vs. OCP data as compared to without inhibitor, indicating that the studied inhibitors are mixed type and dominant towards cathodic nature^48^.

#### Potentiodynamic polarization (PDP)

**Figure 7:**
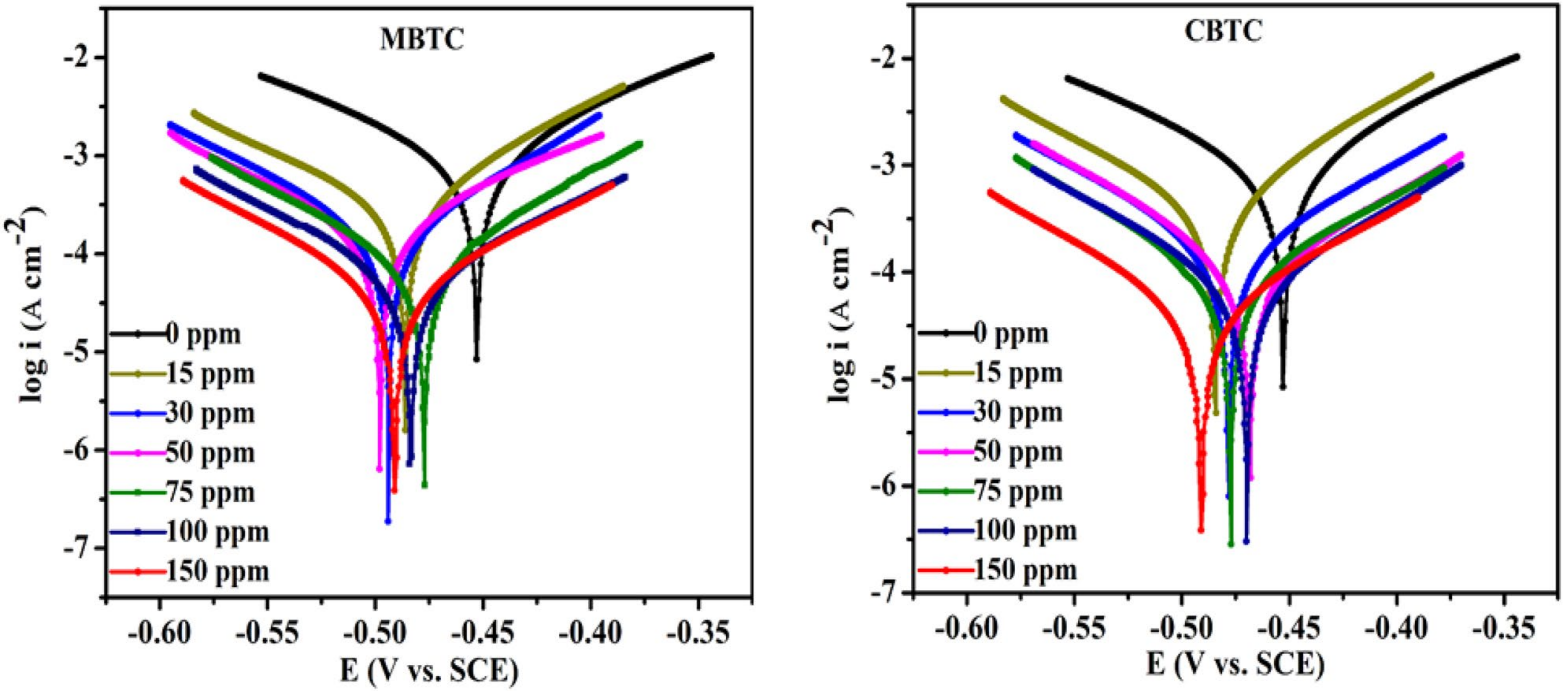
Tafel plot for MBTC and CBTC carbohydrazide Schiff base inhibitors.

PDP is a significant electrochemical method to know about inhibition efficiency of inhibitor. PDP gives value of corrosion current and corrosion potential of the electrode immersed in corrosive solution. Here. PDP curves were plotted to know the impact of various dosages of carbohydrazide Schiff base inhibitor at MS/15% HCl interface as shown in Fig. 7. PDP variables such as, corrosion potential ($${E}_{corr})$$, anodic and cathodic slopes $$({\beta }_{\mathrm{a}}$$ & $${\beta }_{\mathrm{c}}$$), and corrosion current density were observed from PDP curves and arranged in Table 5. The efficiency of corrosion prevention was obtained from Eq. (8) below.

**Table 5:** Polarization data of carbohydrazideschiff base inhibitors.

Conc. (ppm)	${E}_{corr}$ (mV/SCE)	${i}_{corr}$ (µA cm^−2^)	${\beta }_{a}$ (mV dec^−1^)	${-\beta }_{c}$ (mV dec^−1^)	IE_T_%
0	− 427	2017 (± 0.204)	279	277	–
MBTC					
15	− 477.11	399.08 (± 0.021)	89.97	126.78	80.21 (± 0.0015)
30	− 483.62	234.42 (± 0.301)	119.98	121.60	88.37 (± 0.0002)
50	− 483.92	133.09 (± 0.152)	99.06	91.86	93.40 (± 0.0004)
75	− 486.14	91.46 (± 0.067)	86.06	100.47	95.46 (± 0.0034)
100	− 490.84	69.82 (± 0.038)	108.71	101.12	96.53 (± 0.0047)
150	− 497.83	51.51 (± 0.024)	103.43	96.58	97.46 (± 0.0025)
CBTC					
15	− 468.54	499.48 (± 0.028)	86.88	112.78	75.23 (± 0.0022)
30	− 470.02	354.89 (± 0.019)	102.22	138.17	82.85 (± 0.0007)
50	− 473.58	185.02 (± 0.016)	102.91	97.75	90.82 (± 0.0019)
75	− 477.41	124.25 (± 0.015)	102.69	89.77	93.83 (± 0.0045)
100	− 478.44	102.28 (± 0.030)	104.94	96.20	94.92 (± 0.0006)
150	− 484.38	87.56 (± 0.0132)	97.82	98.30	95.65 (± 0.0075)

8 $${\%I.E}_{(T)}=\frac{{i}_{corr}^{(inhi)}}{{i}_{corr}}*100$$

It is noticed that by increase of the conc. of MBTC and CBTC, there is a gradual decrease of ${i}_{corr}$ value. This observation shows the improvement of a protection film on the MS surface by the adsorption of inhibitor molecules that decreases the dissolution of MS surface. This process is more effective with the increment in the conc. of MBTC and CBTC and reaches maximum at 150 ppm 97.46% and 95.65%, individually. Furthermore, PDP shows very less shift in ${E}_{corr}$ data ($less than$ 85 mV) towards more negative side compared to the uninhibited MS, indicating that both inhibitor molecules control anodic as well as cathodic reactions and act as mixed inhibitor. In addition the shift in ${E}_{corr}$ values and the variation of ${\upbeta }_{\mathrm{a}}$ and ${\upbeta }_{\mathrm{c}}$ values with inhibitor concentration w.r.t. without inhibitor also suggested that studied inhibitors are mixed type inhibitors. From Table 5, the data of ${\upbeta }_{\mathrm{a}}$ and ${\upbeta }_{\mathrm{c}}$ do’nt show regular trend which indicate that both physisorption and chemisorption plays an important role in the corrosion inhibition^49^.

#### EIS analysis

**Figure 8:**
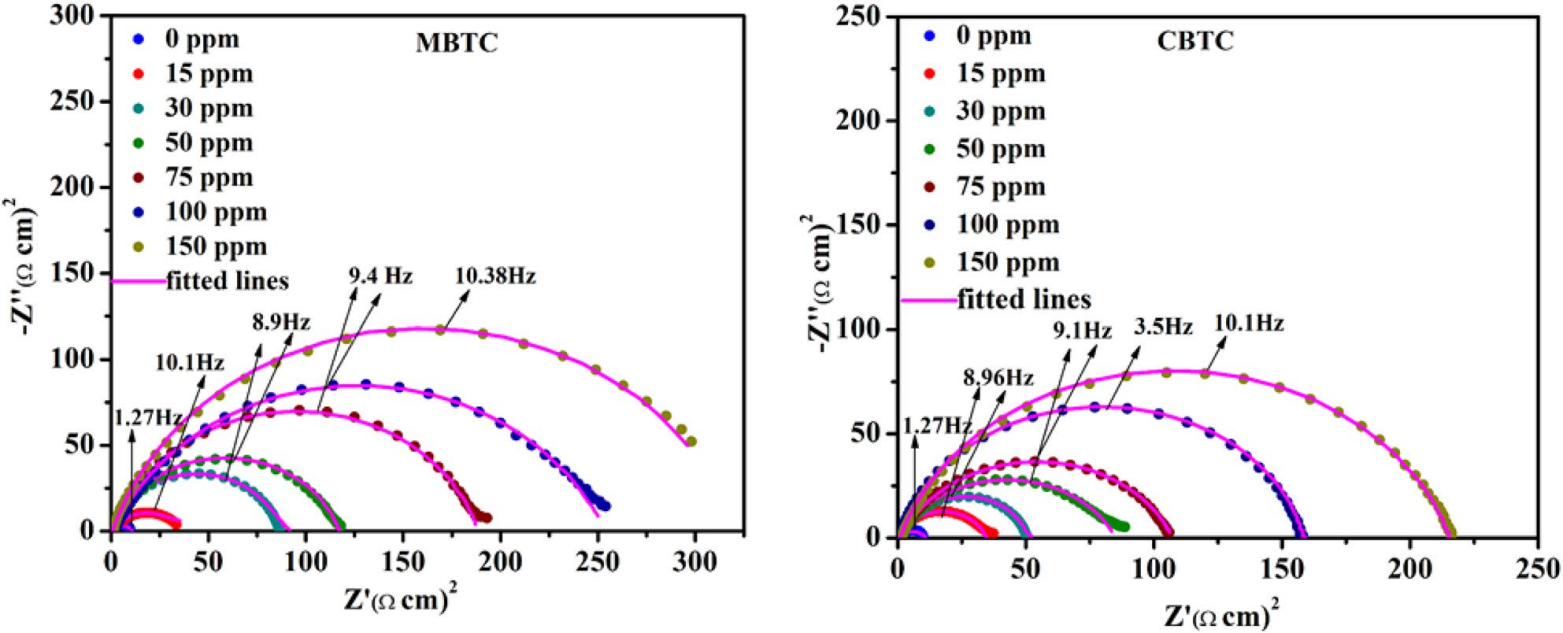
Nyquist plots for MBTC and CBTC carbohydrazide Schiff base inhibitors.

**Figure 9:**
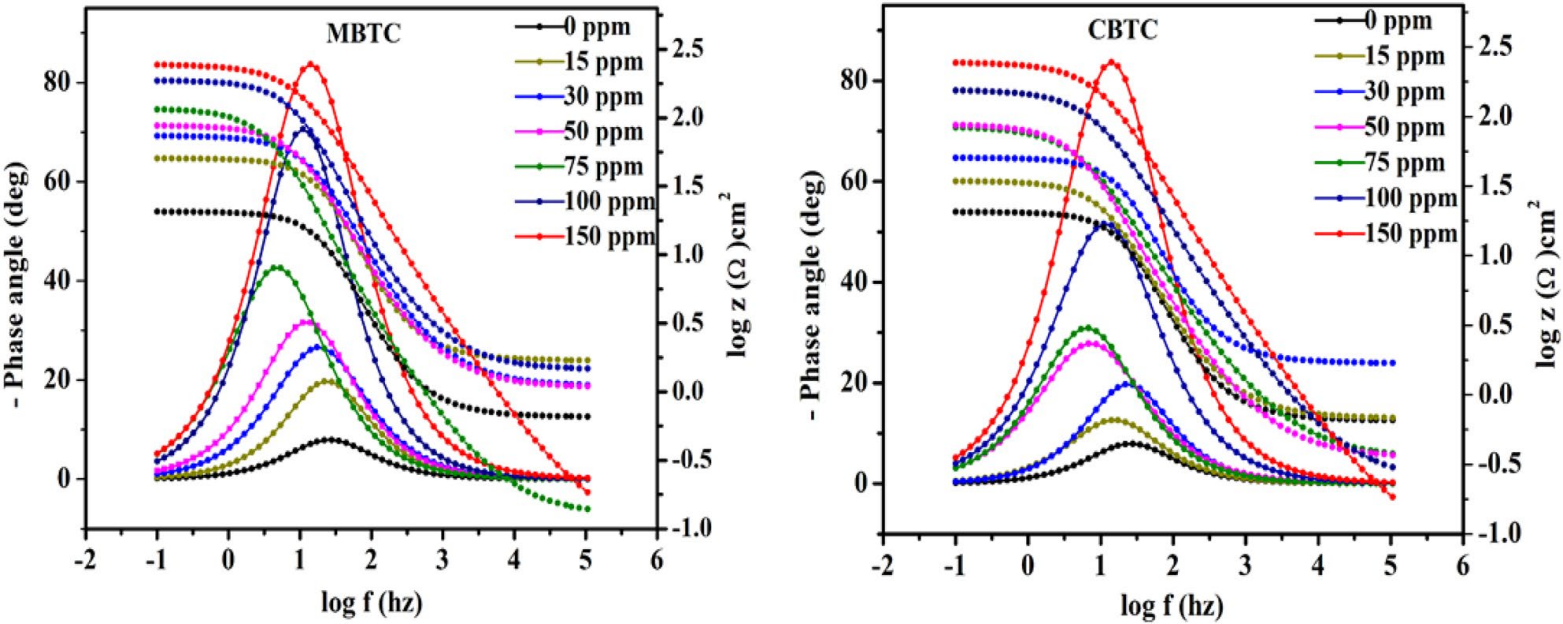
Bode plot for MBTC and CBTCcarbohydrazide Schiff base inhibitors.

**Figure 10:**
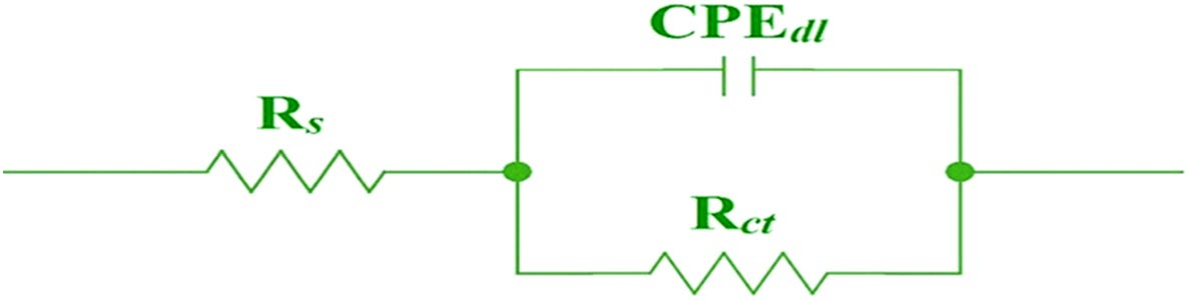
Equivalent circuit used for fitting EIS graphs.

EIS is used to correctly measure the inhibition efficiency of an inhibitor. The Nyquist and Bode plots were obtained without and with MBTC and CBTC inhibitors as depicted in Figs. 8 and 9, respectively. Nyquist semicircles were fitted against electrical circuit, shown in Fig. 10. This circuit consist of solution resistance $$({R}_{S})$$, a double layer capacitance ($${C}_{dl})$$ and a charge transfer resistance $${(R}_{ct})$$. The obtained data are shown in Table 6. The obtained Nyquist plots have depressed semi-circles with a single loop, suggesting single time constant participation in the EIS spectra^50^. The depression in semi-circle is because of inhomogeneity of electrode surface. The rise in diameter of capacitive loops with an increment in carbohydrazide Schiff base inhibitor concentration suggests an increase in the charge transfer resistance as a result of inhibitor adsorption on MS surface. The impedance efficiency was obtained by utilizing the accompanying equation:

9 $${\%I.E}_{NY}=\frac{{R}_{ct}^{\left(inhi\right)}-{R}_{ct}^{\left(acid\right)}}{{R}_{ct}^{inhi}}*100$$

where,${R}_{ct}^{\left(inhi\right)}$ and ${R}_{ct}^{\left(acid\right)}$ are the polarization resistance data in the inclusion along with exclusion of an inhibitor individually.

**Table 6:** EIS data for carbohydrazideschiff base inhibitors.

Conc. (ppm)	${R}_{S}$ (Ω cm^2^)	${R}_{ct}$ (Ω cm^2^)	Y_0_ (µF cm^−2^)	n	${C}_{dl}$ (µF cm^2^)	IE_NY_%
0	0.6561 (± 0.012)	10.42 (± 0.300)	764 (± 0.573)	0.8744 (± 0.001)	381.58 (± 0.504)	–
MBTC						
15	0.8432 (± 0.022)	37.22 (± 0.551)	246.88 (± 0.349)	0.8605 (± 0.002)	115.42 (± 0.442)	72.00 (± 0.567)
30	0.8809 (± 0.017)	62.89 (± 0.315)	238.73 (± 0.441)	0.8333 (± 0.002)	103.10 (± 0.365)	83.43 (± 0.342)
50	0.8870 (± 0.002)	105.18 (± 0.203)	223.43 (± 0.344)	0.8169 (± 0.003)	96.39 (± 0.278)	90.09 (± 0.144)
75	0.4516 (± 0.003)	187.00 (± 1.09)	172.74 (± 0.865)	0.8167 (± 0.001)	79.94 (± 0.675)	94.42 (± 0.732)
100	0.6890 (± 0.019)	256.00 (± 0.675)	140.75 (± 0.752)	0.8097 (± 0.002)	64.45 (± 0.867)	95.92 (± 0.629)
150	0.6246 (± 0.012)	320.67 (± 1.331)	131.06 (± 1.291)	0.8032 (± 0.002)	60.28 (± 0.652)	96.75 (± 0.530)
CBTC						
15	0.6785 (± 0.011)	33.82 (± 0.211)	704.56 (± 1.191)	0.8579 (± 0.001)	379.40 (± 0.761)	69.18 (± 0.134)
30	0.6918 (± 0.014)	48.80 (± 0.453)	690.71 (± 0.672)	0.8415 (± 0.003)	364.74 (± 0.870)	78.64 (± 0.771)
50	0.3516 (± 0.010)	84.67 (± 1.213)	312.86 (± 0.342)	0.8288 (± 0.004)	276.91 (± 0.528)	87.69 (± 0.349)
75	0.8840 (± 0.025)	137.89 (± 0.567)	288.57 (± 0.202)	0.8189 (± 0.002)	141.42 (± 0.923)	92.44 (± 0.166)
100	0.6870 (± 0.002)	157.09 (± 0.214)	264.47 (± 1.012)	0.8176 (± 0.001)	130.07 (± 0.591)	93.36 (± 0.459)
150	0.8970 (± 0.035)	214.48 (± 1.092)	189.14 (± 0.623)	0.8070 (± 0.003)	87.98 (± 0.491)	95.14 (± 0.152)

As the inhibitor concentration increases, ${C}_{dl}$ reduces and ${R}_{ct}$ increases as represented in Table 6, the results confirmed the high degree of adsorption of carbohydrazide Schiff base at MS/HCl interface by sharing of non-bonding heteroatoms electrons of inhibitor molecules to the vacant d-orbital of Fe. MBTC shows maximum 96.75% inhibition efficiency at 150 ppm against 320.67 Ωcm^2^ ${R}_{ct}$ value, Whereas CBTC shows maximum 95.14% against 214.48 Ω cm^2^ ${R}_{ct}$ value with ${R}_{ct}$ value. It confirms that MBTC has better inhibition performance against the 15% HCl corrosive medium. The value of ${C}_{dl}$ was obtained by equation

10 $${C}_{dl}={\left({Y}_{0}{R}_{ct}^{inhi)1-n}\right)}^{1/n}$$

where (Y_0_) for proportional factor and (n) for phase shift and it is measured for the surface inhomogeneity.

In Bode plots continuing increment in the phase angle with concentration up to 150 ppm is related to the development of surface coverage by the inhibitors MBTC and CBTC molecules. The maximum height in the phase angle towards − 90°, the existence of single maxima demonstrates presence of one time constant related to electrical double layer at the surface solution interface^52^.

The inhibition efficiency calculated by gravimetric, PDP, & EIS methods for MS in presence and absence of inhibitors in 15% HCl solution are in good agreement to each other. The high corrosion inhibition proficiency value of both studied MBTC and CBTC even at lower concentration suggested that MBTC and CBTC are good corrosion inhibitors for MS in acidic solution.

### Surface morphology study

#### FESEM analysis

**Figure 11:**
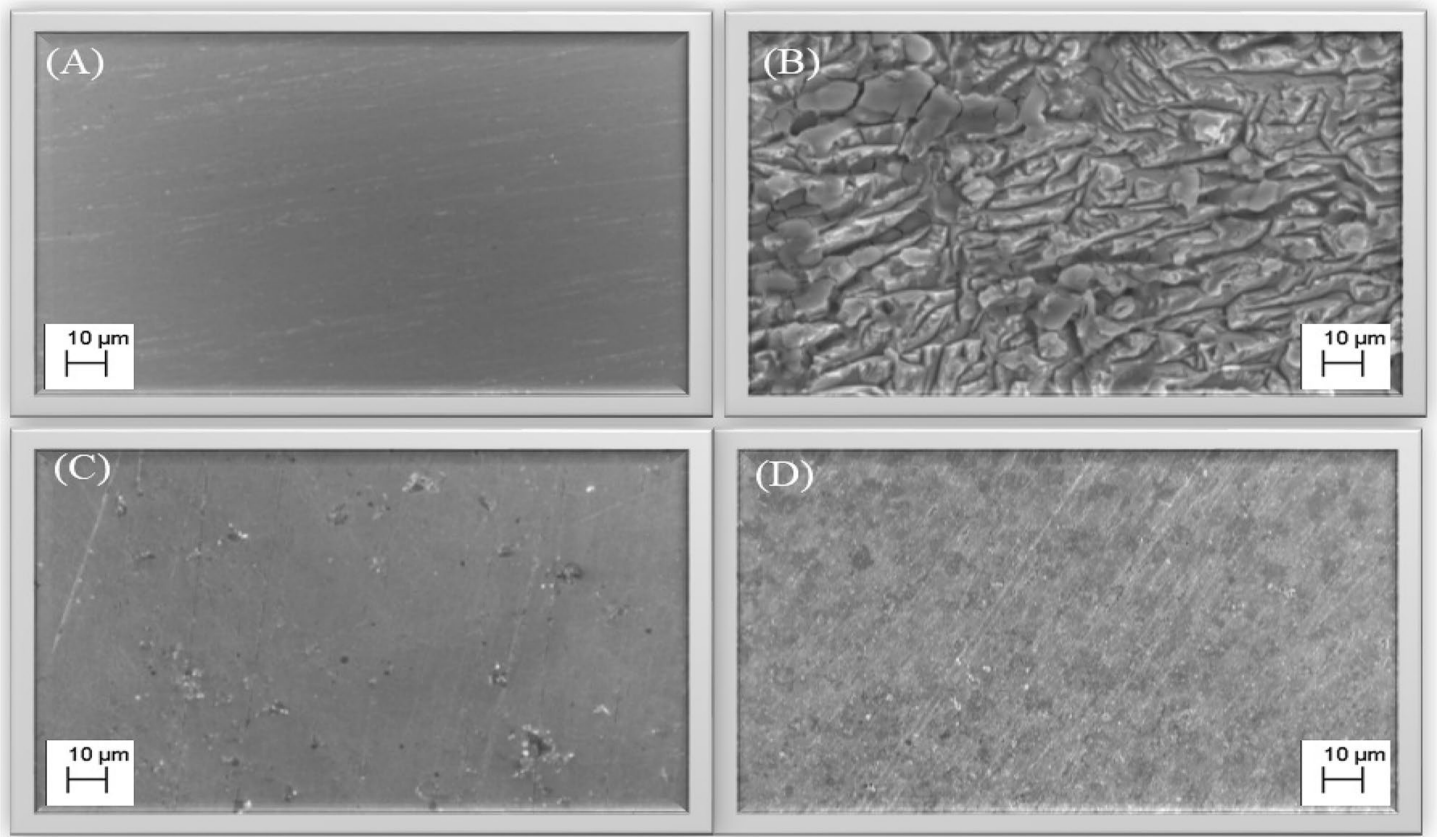
FESEM pictures of polished, blank, MBTC and CBTC inhibitor treated surface (**A**–**D**) respectively.

The surface morphology of polished MS, after 6 h of immersion in 15%HCl solution in the absence and presence of 150 ppm for MBTC and CBTC inhibitors are depicted in Fig. 11. From Fig. 11B, it is observed that the metallic surface is extremely corroded in HCl solution without inhibitor whereas polished sample (Fig. 11A) is smooth due to minor corrosion. However, Fig. 11C and D containing inhibitors show very smooth MS surface and it contain less cracks and pits due to adsorption of MBTC and CBTC inhibitors, respectively, at MS surface. Consequently, these inhibitors demonstrated that MS is protected against corrosion by forming protective layer of inhibitor at the surface of mild steel^53^.

#### EDX analysis

**Figure 12:**
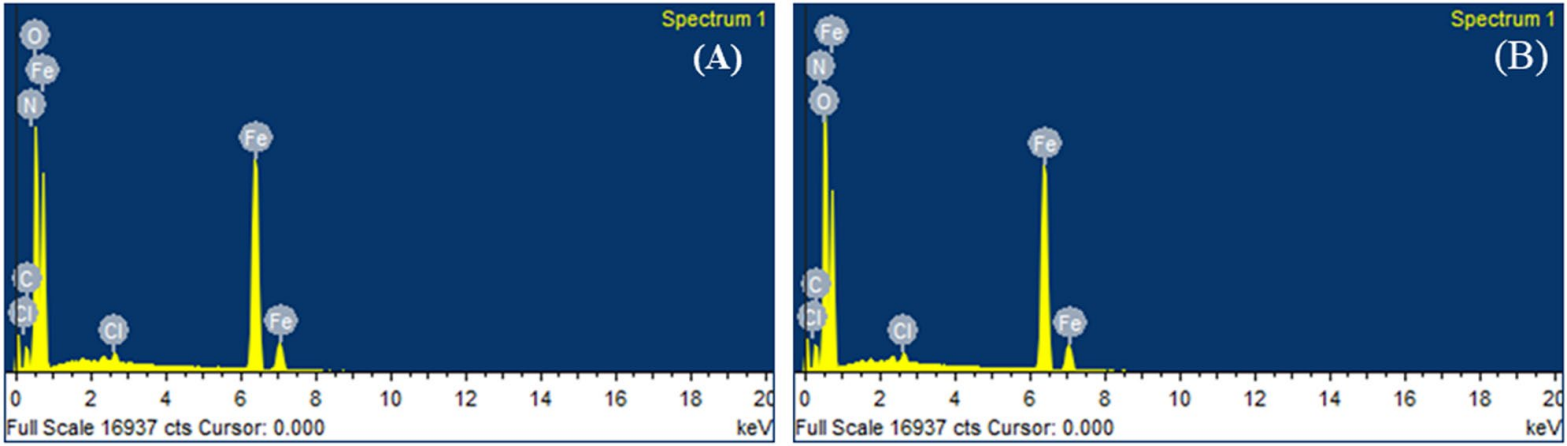
EDX spectra for (**A**) with MBTC (**B**) with CBTC inhibitors.

The EDX images of MS surface containing MBTC and CBTC are shown in Fig. 12A,B, respectively. The presence of C, N, O, and Cl, confirms the presence of adsorbed inhibitor layer on MS surface^54^.

#### AFM analysis

**Figure 13:**
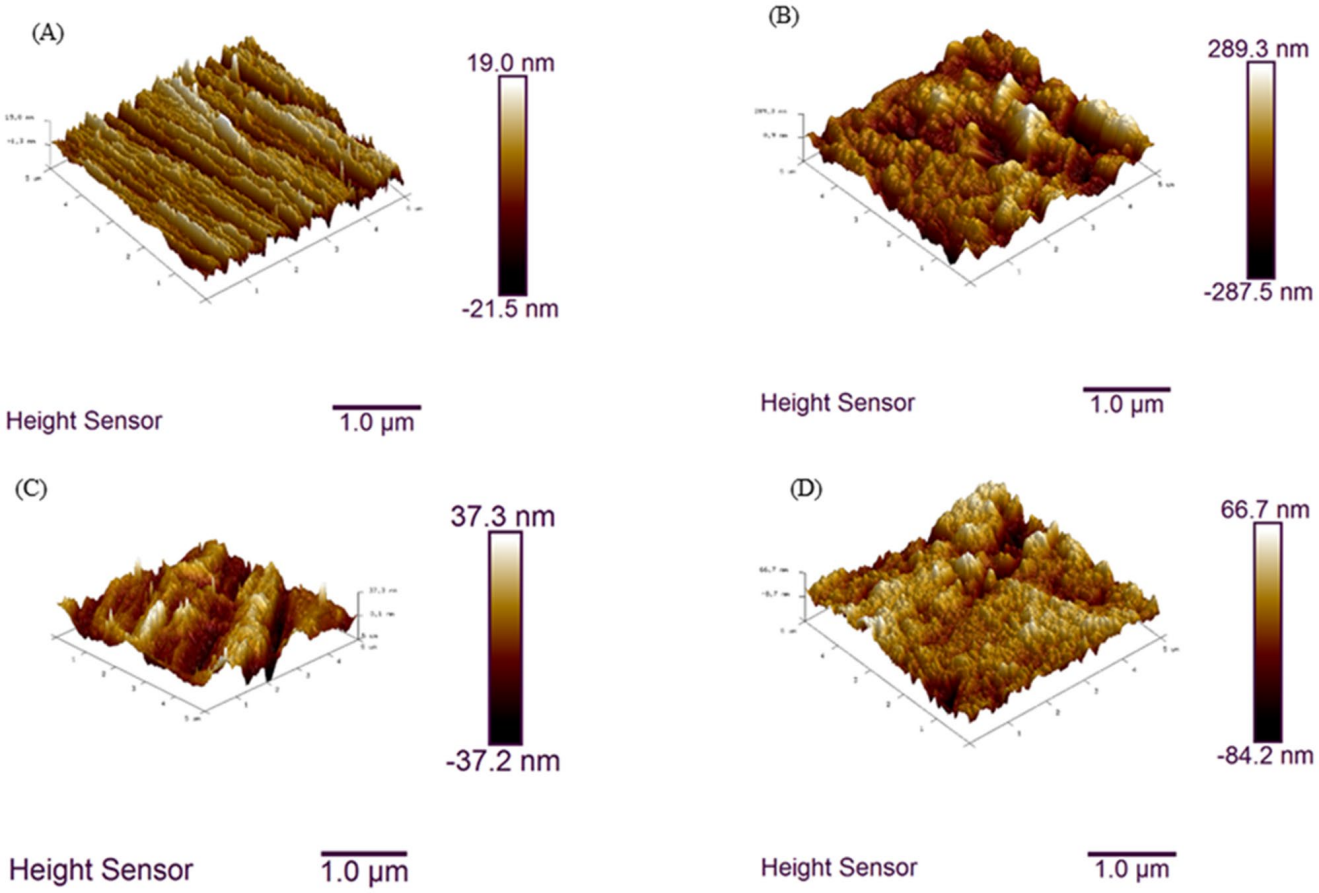
AFM images for polished (**A**), blank (**B**), with inhibitors MBTC (**C**), and CBTC (D).

AFM analysis is an important technique for 3-D representation of surface with appropriate height profile of inhibitors. It consists of the important parameter of average roughness (R_max_), average of maximum height (R_a_), and the greatest separation among the maximum point and minimum points on the profile roughness (R_q_). The AFM of samples are shown in Fig. 13. The result for the height profile for polished surface (Fig. 13A) are R_max_ = 57.3 nm, R_q_ = 6.07 nm, R_a_ = 4.92 nm. For blank sample (Fig. 13B) R_max_ = 567 nm, R_q_ = 77.9 nm, R_a_ = 62.6 nm. From Fig. 13C the surface with MBTC containing R_max_ = 107 nm, R_q_ = 10.3 nm, R_a_ = 8.14 nm and Fig. 13D, in presence of CBTC, R_max_ = 156 nm, R_q_ = 20.0 nm, R_a_ = 15.4 nm. The lower value of roughness in presence of inhibitors is because of the adsorption of inhibitor molecules on the MS surface^55^.

#### XPS analysis

**Figure 14:**
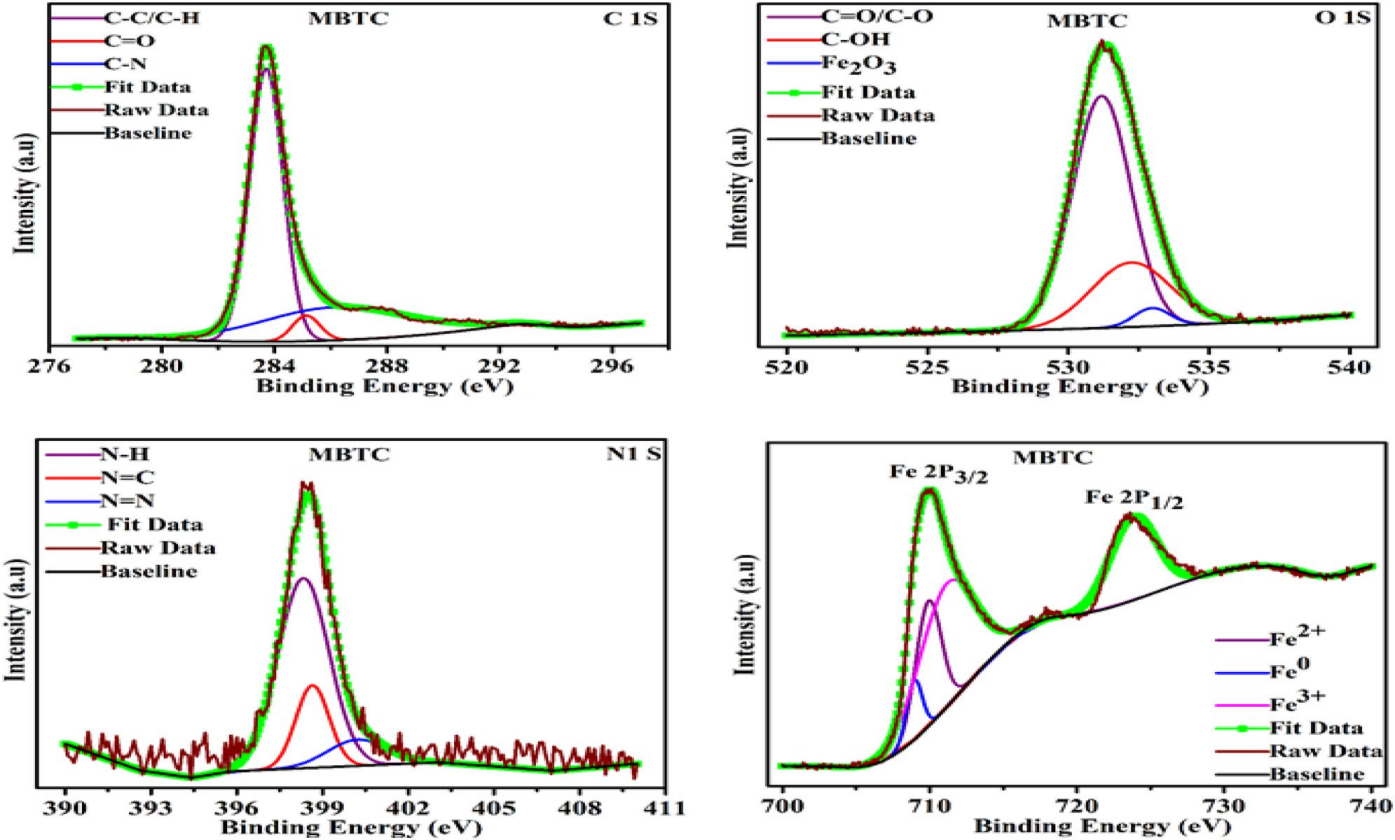
XPS spectra for deposited layer for MBTC inhibitors.

**Figure 15:**
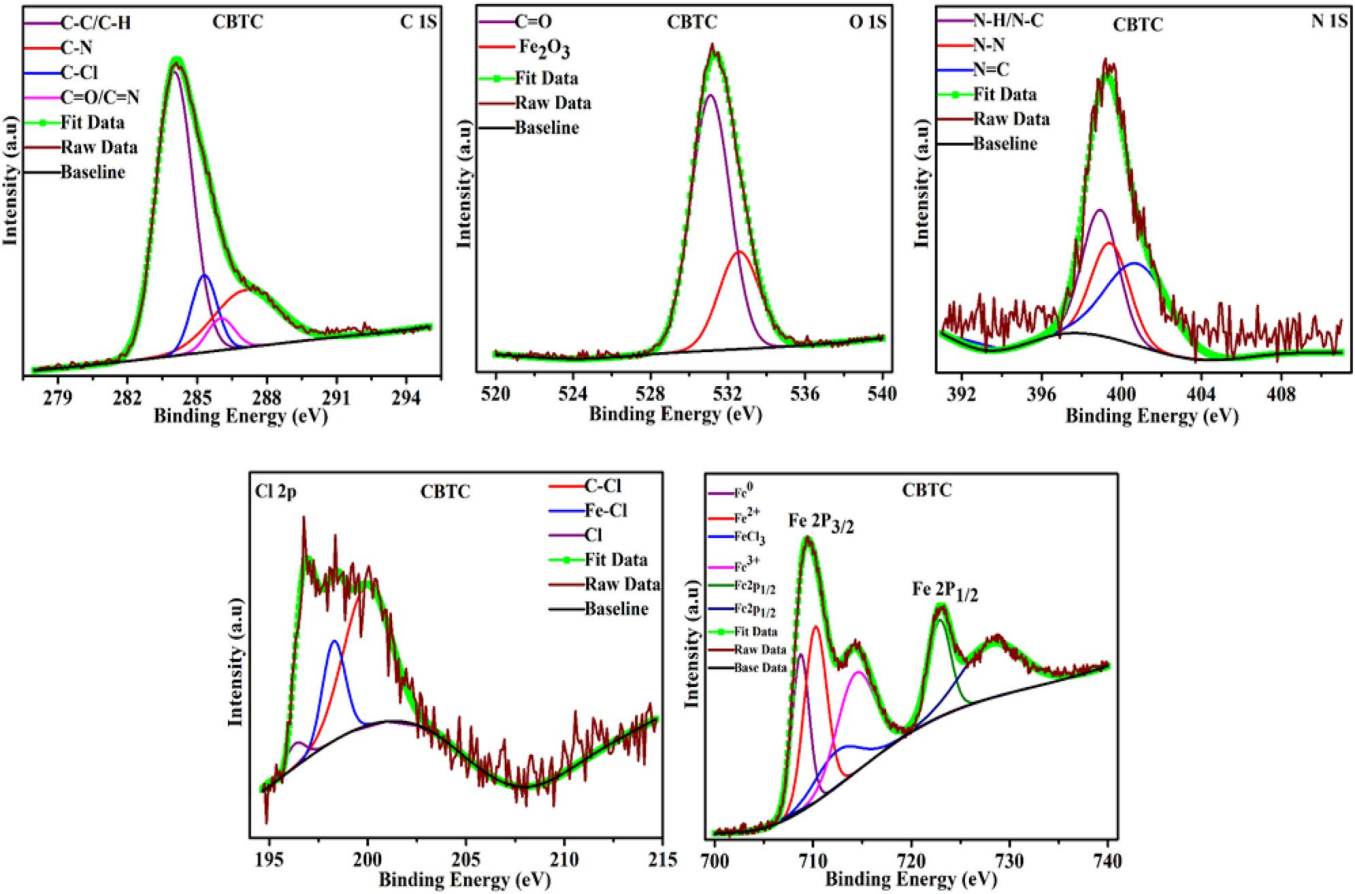
XPS spectra for deposited layer for CBTC inhibitors.

XPS is used to know the composition of adsorbed inhibitor layer on MS surface. Result of XPS analysis of MBTC and CBTC inhibitor are shown in Figs. 14 and 15 respectively.

In Fig. 14, three peaks of C1s spectrum with binding energies of 283.76, 285.05, and 285.80 eV are assigned to the C–C or C–H, C=O and C–N bonds respectively^56,57^. The O1s peaks at 531.16 eV, 532.30 eV along with 533.09 eV, respectively, connected to O=C or C–O, C–OH, and Fe_2_O_3_^57,58^. Three peaks in the N1s spectrum, at 398.21, 398.76, and 400.30 eV are associated to N–H, N=C, and N=N, respectively^54,58^. The Fe 2P_1/2_ spectrum contains three peaks at 708.93, 710.20, 711.48 eV that are linked to metallic iron, Fe^2+^, and Fe^3+^ whereas the spectrum Fe 2P_3/2_ only one peak at 723.72eV^33,58,59^.

Four peaks of CBTC inhibitor adsorbed on the MS surface were observed in the C1s spectrum in Fig. 15.The peak at 284.07 eV corresponds to C–C or C-H aromatic bond. The peak for the C-atom in the C=O and C=N bonds are at 286.07 eV^59^. The C–Cl bond and the C–N bond at 285.29 and 287.03^54^. The peak for the Fe_2_O_3_ bond can be seen in the O1s spectrum at 532.77 eV, whereas the peak for C=O is at 531.19 eV^60^. The N1s spectrum represents three peaks at binding energy 398.74, 399.18, and 400.64 eV attributed for NH or NC bond, N–N bond and N=C bond of the inhibitor CBTC^3,61^. There are three peaks in the Cl 2P peak, with the peak at 196.41 eV for chlorine ion of the HCl solution, the peak at 198.21 eV representing the Fe-Cl bond, and the peak at 200.08 eV describing the C–Cl bond of the organic inhibitor^33,56^. The Fe 2P1/2 spectrum comprises four peaks for metallic iron, Fe^2+^, FeCl_3_, and Fe^3+^ at 708.70, 710.36, 713.28, and 714.56 eV, but Fe 2P3/2 has two peaks at 723.05 and 728.75 eV^62,63^.

### Theoretical analysis

#### DFT results

**Figure 16:**
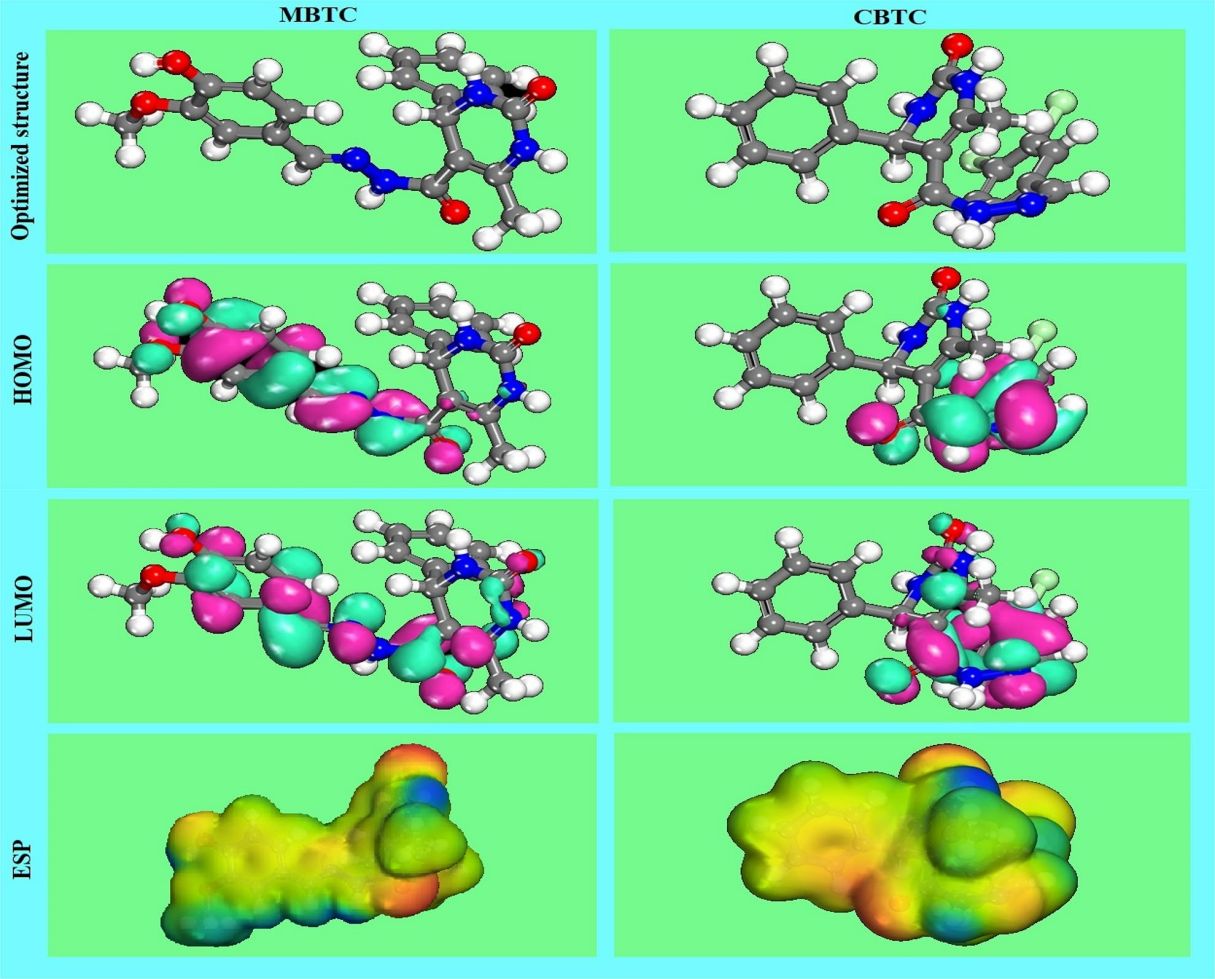
Molecular images of the MBTC and CBTC with optimized structures in HOMO, LUMO, and ESP.

The optimized structures, HOMO/LUMO frontier orbitals as well as the molecular electrostatic potential (ESP) are represented in Fig. 16. Table 7 represents the main orbital parameters calculated for the MBTC and CBTC inhibitors.

**Table 7:** Calculated theoretical parameters for MBTC and CBTC molecules.

Theoretical parameters	MBTC	CBTC
*E*_HOMO_ (eV)	− 5.134	− 5.656
*E*_LUMO_ (eV)	− 2.186	− 2.705
∆E (*E*_HOMO_ − *E*_LUMO_) (eV)	2.948	2.951
I (eV)	5.134	5.656
A (eV)	2.186	2.705
χ (eV)	3.660	4.180
η (eV)	1.474	1.475
σ (eV^−1^)	0.678	0.677
∆N	1.132	0.955
∆E_back-donation_	− 0.368	− 0.368
Dipole magnitude	9.562	4.126

The least amount of energy needed to excite an electron in a molecule is known as the energy gap (∆E = *E*_HOMO_ − *E*_LUMO_). The complex produced on the metallic surface is very stable if the value of ∆E is low, indicating that the molecule's inhibitory effectiveness is strong. Hence, decreasing the value of ∆E enhances the molecule's reactivity, which makes adsorption easier and boosts the inhibitor's effectiveness^64^. In this work, the difference between the energy levels ∆E of MBTC (2.948 eV) and CBTC (2.951 eV) shows that MBTC has better corrosion-inhibiting properties than CBTC. Often the dipole moment (μ) because of the polarity of an inhibitor and is associated with the inhibitory capacity^65^. A high value of the μ results in significant inhibitory efficacy and increases the interaction (adsorption) of inhibitor compounds with a metal surface^42^.Table 7 shows that the μ of the MBTC (9.562 Debye) is greater than μ of the CBTC (4.126 Debye). As well as, both inhibitors are greater than that of the H_2_O molecule (1.88 Debye), which explains the significant inhibitory efficacy of the MBTC inhibitor. According to Table 7, the data of ∆N for both inhibitor is positive and greater than 3.6 (N > 3.6), which shows that electrons are transferred from both inhibitors studied to iron, promoting the development of coordination bond and thereby helping the formation of an adsorbed layer against corrosion.

To determine the active sites accountable for nucleophilic and electrophilic attacks, decided to use ESP surfaces as a useful descriptor. Figure 16 shows the ESP of the MBTC and CBTC molecules inhibitors study, the blue color shows the positive zones with nucleophilic reactivity, whereas yellow and red colors represent the negative zones with electrophilic reactivity.

As displayed in Fig. 16, the two inhibitors studied have 4 oxygen atoms possible electrophilic attack sites for the MBTC molecule and 2 possible electrophilic attack sites for the CBTC molecule. On the other hand, the two aromatic rings for the two compounds studied have a negative region. There are also on the two molecular structures of our inhibitors the 4 nitrogen atoms which are 4 other possible sites for the electrophilic attack. From the calculated zones of ESP, we notice that the potential negative zones possible are located on the electronegative atoms (N and O) and the unsaturated double bonds along with potential positive zones possible were located around the hydrogen atoms.

#### MC and MD simulations

The study of the adsorption behavior of the MBTC and CBTC molecules were examined on the surface of MS using MC and MD simulations^66–68^.

**Figure 17:**
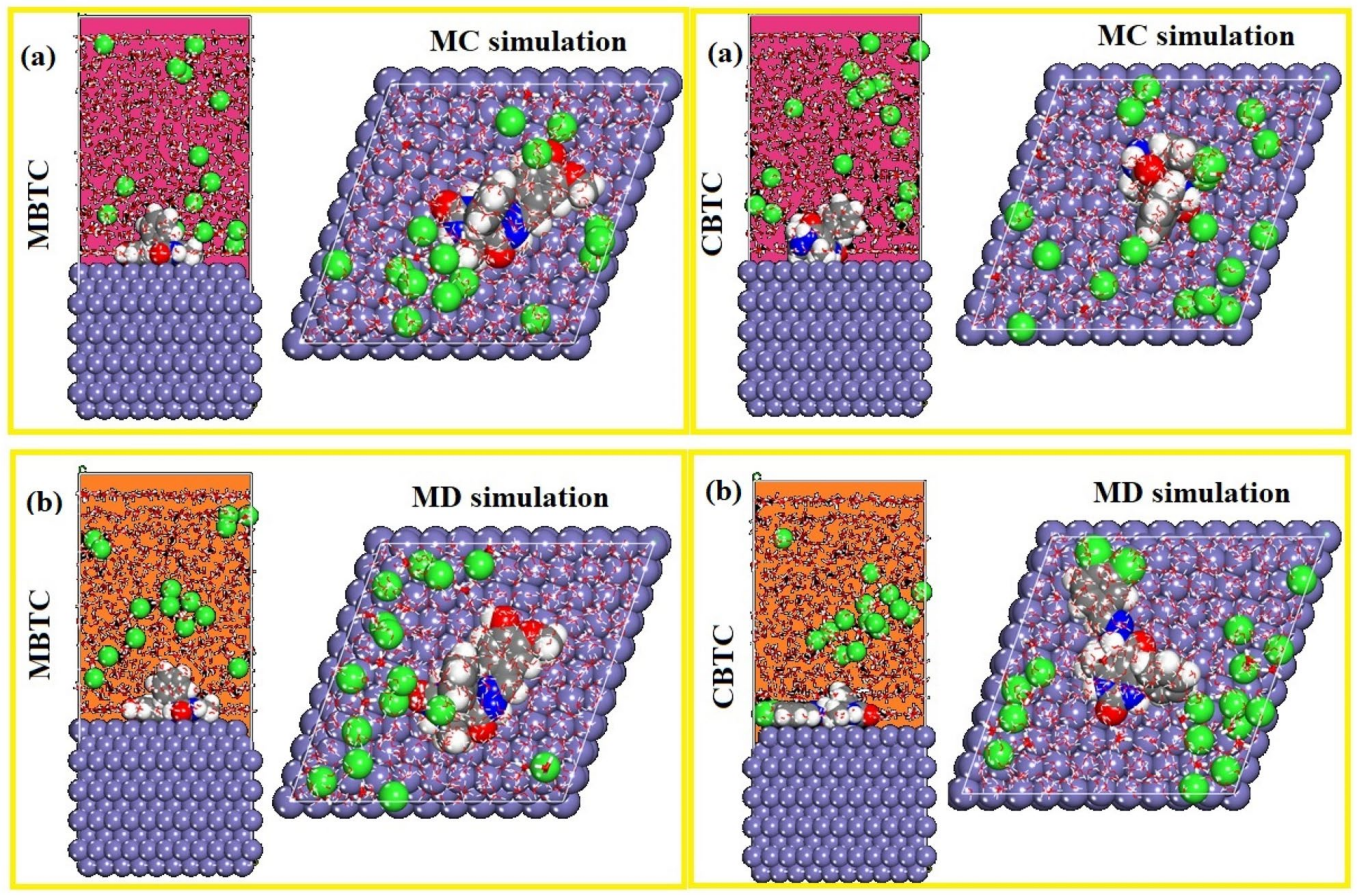
Results of MC (**a**) and MD (**b**) simulations: The equilibrium configurations of MBTC and CBTC molecules adhering to the surface of iron (110).

Figure 17shows the adsorption equilibrium configurations for the two MBTC and CBTC molecules on the Fe (110) surface, thus two inhibitory molecules are adsorbed on the Fe (110) surface in almost parallel mode position (almost horizontal), indicating that the adsorbed inhibitor molecules cover the Fe surface well by developing a horizontal protective layer on the steel surface, which confirms the strong interactions among the heteroatoms (O, N) and the conjugated system of these inhibitors and atoms of iron.

**Figure 18:**
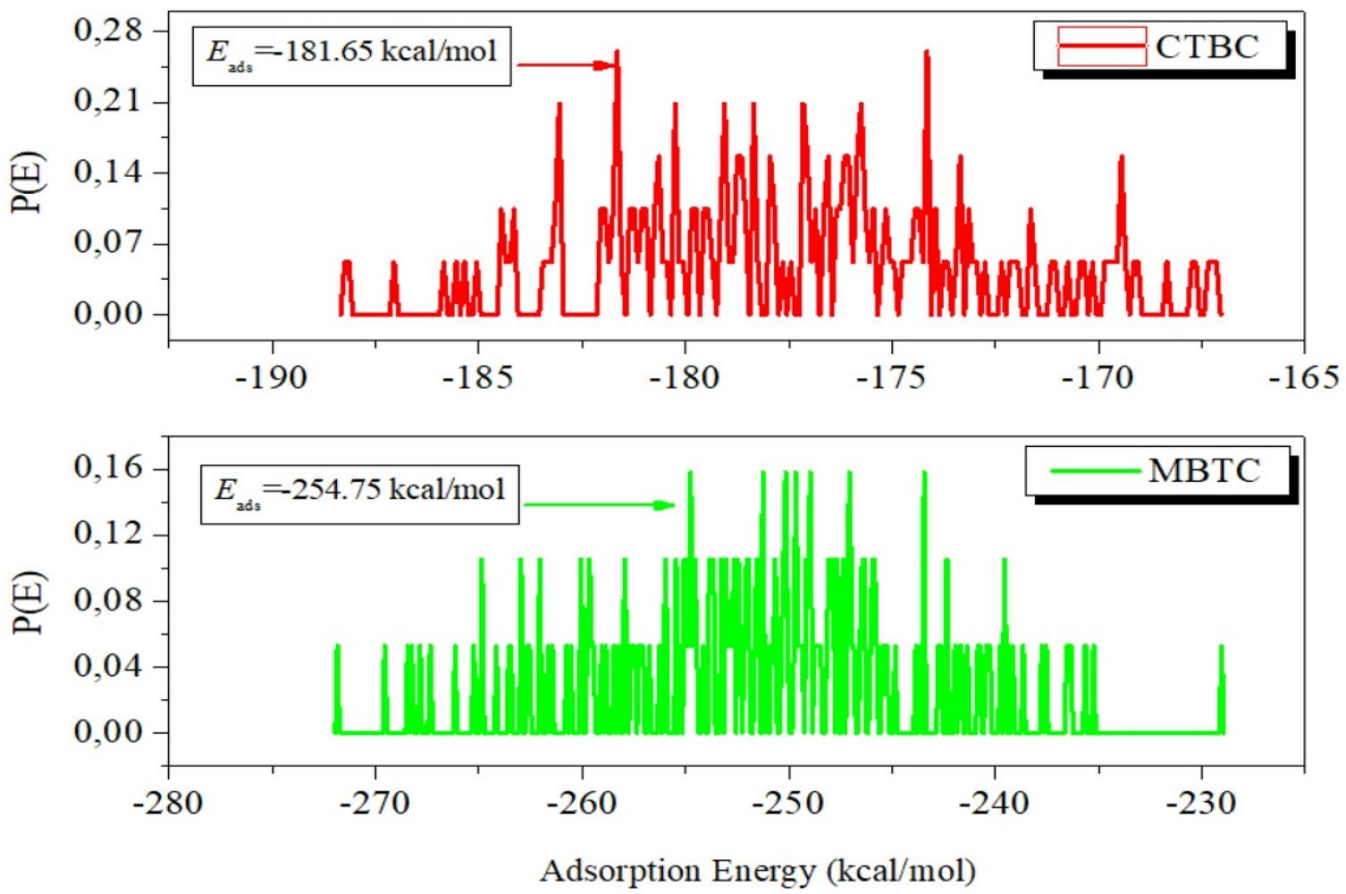
Distribution of the *E*_ads_ of MBTC and CBTC:MC simulations of the molecules on Fe (110) plane.

Figure 18 displays the adsorption energy (*E*_ads_) that was calculated by MC simulations of the MBTC and CBTC molecules with the surface of Fe (110). The compounds investigated are listed in the following order according to their effectiveness at inhibiting growth: MBTC > CBTC.

**Figure 19:**
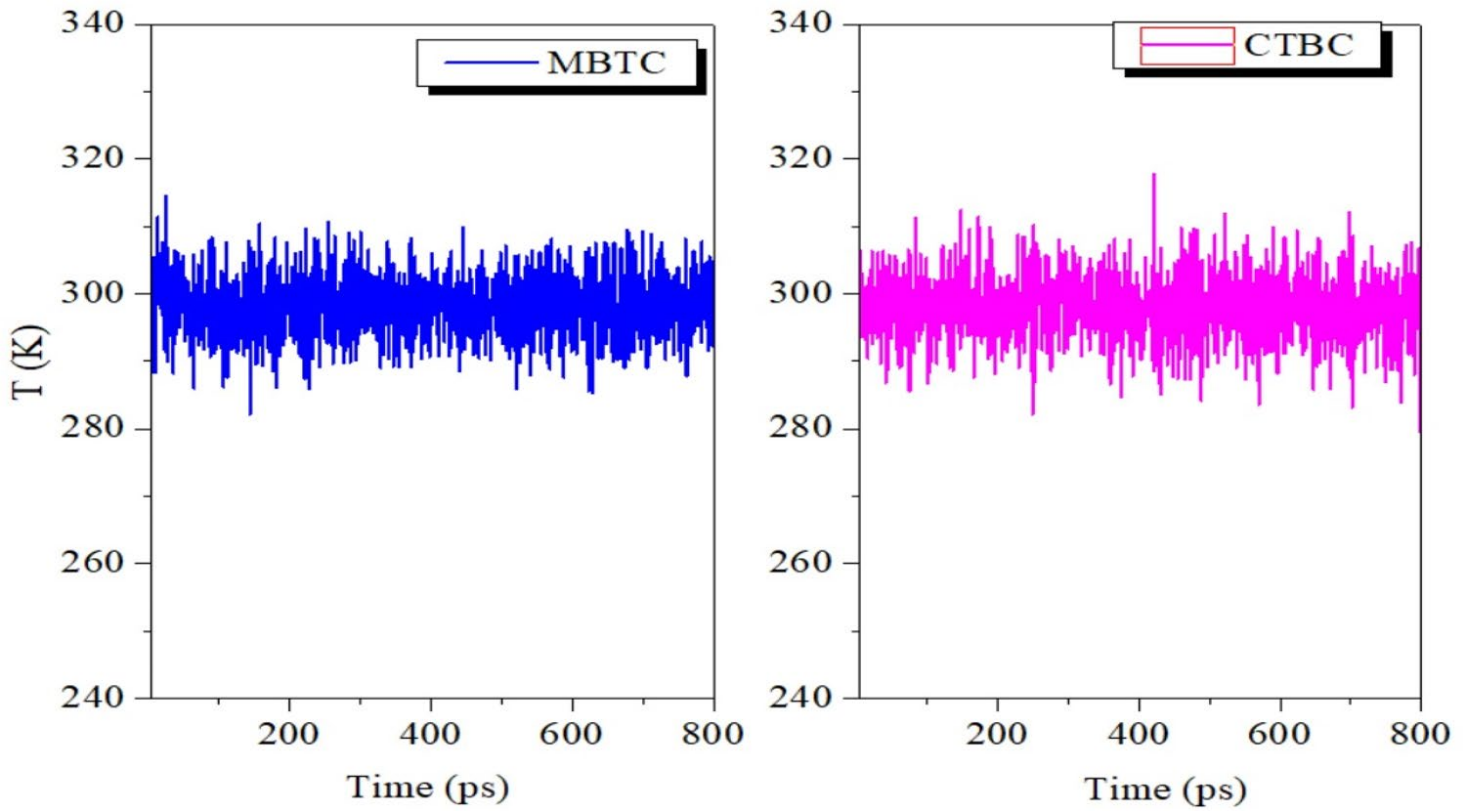
Temperature fluctuation at 298 K during the MD run of MBTC and CBTC molecules on the Fe(110) plane.

The most energetic stable positions of inhibitors are found by studying the temperature variations in MD simulation analyses. The little temperature drift in Fig. 19 indicates that the MD of our system was successful^69,70^.

**Figure 20:**
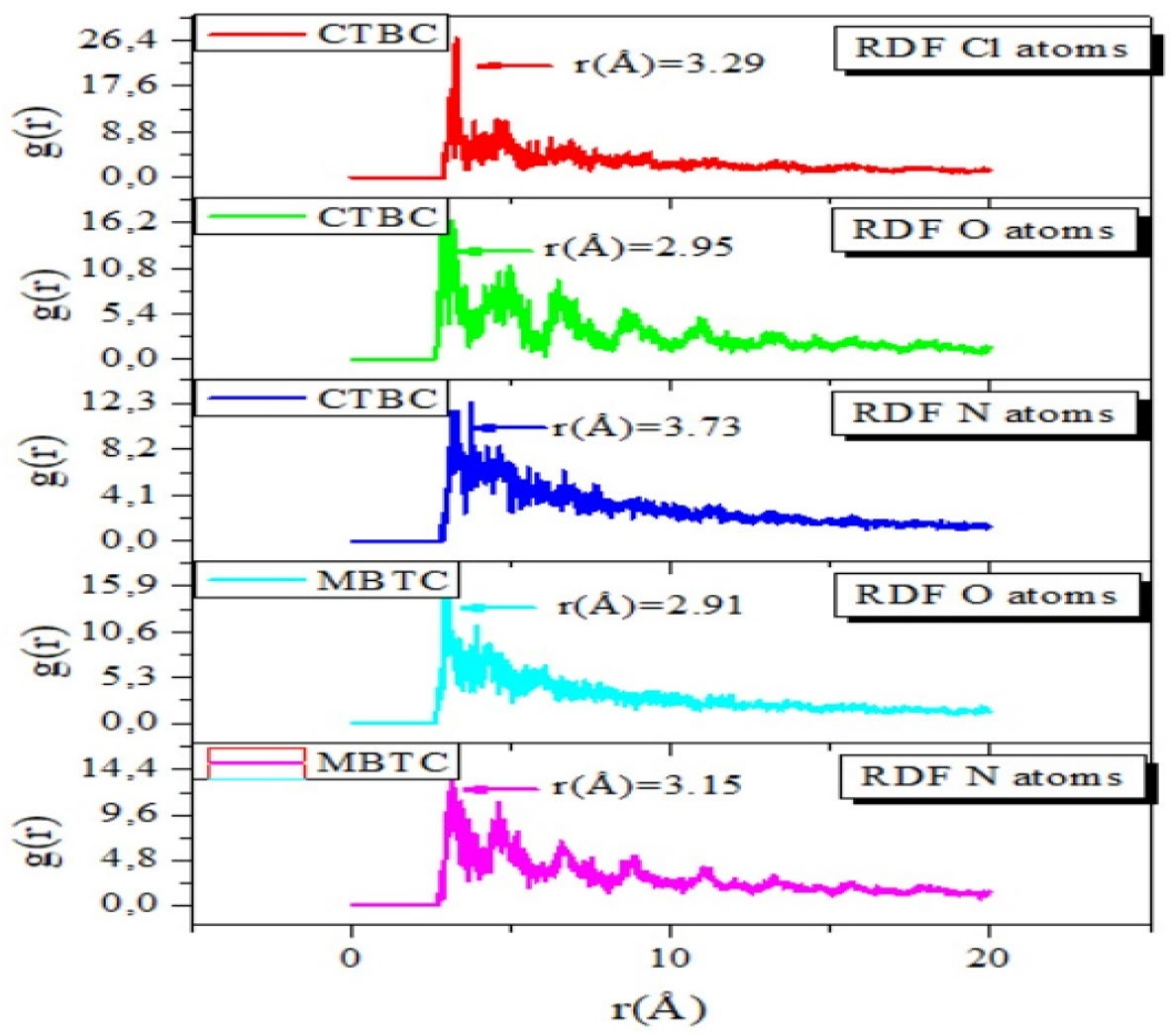
RDF of O, N and Cl atoms of MBTC and CBTC in the simulated corrosion media on the Fe plane via MD.

The bond length among the iron and the atoms of the MBTC and CBTC was determined by utilizing the radial distribution function (RDF) analysis of the MD trajectory in Fig. 20.Through estimating bond length data, different types of bonds formed was determined^39,71^.The type of adsorption activity occurring on the metal is shown as peaks in the RDF plot at specific locations relative to the metal surface^41,72,73^.The mechanism of chemisorption is reflected, when the peak is present between 1 and 3.5, but the RDF peaks are expected for physisorption at distances larger than 3.5^74–76^.

### The inhibition mechanism

**Figure 21:**
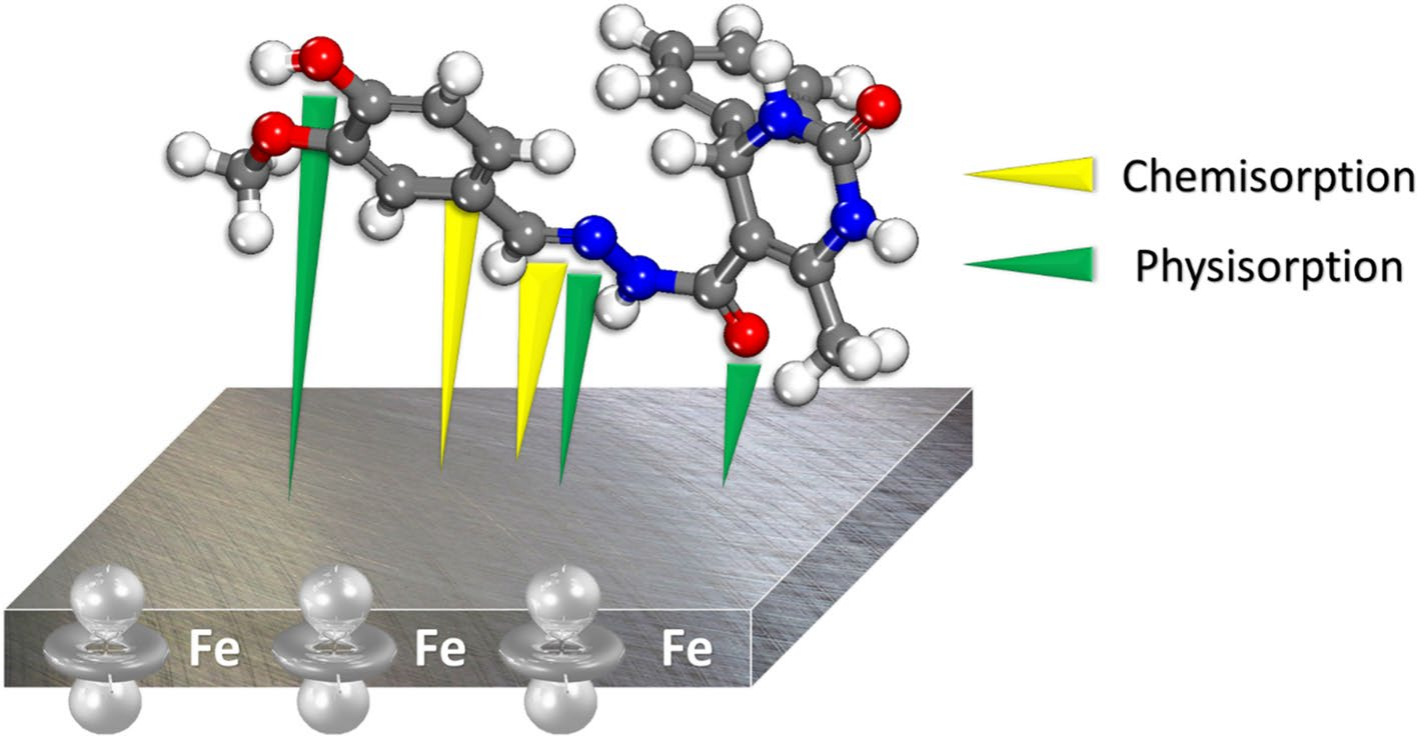
Diagram of the mechanisms of MBTC adsorption on mild steel.

The hypothesized corrosion mechanism of MBTC for MS in 15% HCl solutionis presented in Fig. 21, as determined by the experimental and theoretical investigations. Inhibitor molecules, by virtue of their adsorptive affinity, reject the pre-adsorbed H_2_O molecules and corrosive species, finally adsorb on the surface of MS, therefore sheltering cathodic along with the anodic site^77,78^. This protects the surface against corrosion. In particular, because the compound possesses both a heterocyclic ring and other heteroatoms, as shown by the Mulliken charges, there is an increased predisposition toward surface adsorption^79^. These heteroatoms (O, N) and the conjugated system are able to donate lone pair electrons to the empty d-orbit of the iron atom^80^.

Adsorption has an important role in corrosion prevention because the organic molecules are effectively and efficiently adsorbed on the surface, thus stop further deterioration on the surface of MS. All of the information mentioned in carbohydrazide Schiff bases supports this process. The thermodynamic properties of adsorption and theoretical calculations imply that the adsorption mechanism involves physical as well as chemical adsorption. Hence, carbohydrazide Schiff base adsorption on MS surface is explained by the donor–acceptor interactions, which link the electrons of nitrogen, oxygen, and the aromatic rings of inhibitors to the unoccupied d-orbitals of Fe surface atom. The ability of the inhibitor to specifically bind to the metal's surface and displace water molecules is responsible the corrosion-inhibiting process.

## Conclusion

Both the studied Schiff base carbohydrazide compounds MBTC and CBTC contain pyrimidine as well as aromatic rings, heteroatoms (oxygen and nitrogen) and planar structures through which adsorb on the MS surface and act as efficient corrosion inhibitor. The inhibitors MBTC and CBTC offered efficiency of 97.45% and 95.89%, respectively, at 150 ppm concentration. The efficiency offered by both inhibitors increased on increasing concentration and decreased on increasing temperature. The potentiodynamic polarization measurements concluded that MBTC and CBTC behave as mixed inhibitors. The inhibitors MBTC and CBTC adsorbed as per Langmuir isotherm and mixed adsorption process. The adsorption of inhibitors on MS surface was confirmed by the FESEM, EDX and XPS analysis. The analysis of the DFT-derived descriptors revealed that the molecules' heteroatoms are involved in the adsorption of these inhibitors onto the MS surface. As per MCS and MDS analysis, both inhibitors have a strong interaction, resulting in their adsorption onto the Fe (100) surface plane. This adsorption prevents corrosion activity from the MS surface.

## Data Availability

The authors declare that the data supporting the findings of this study are available within the paper.
